# Genome-wide identification of the DUF668 gene family in cotton and expression profiling analysis of GhDUF668 in *Gossypium hirsutum* under adverse stress

**DOI:** 10.1186/s12864-021-07716-w

**Published:** 2021-05-27

**Authors:** Jieyin Zhao, Peng Wang, Wenju Gao, Yilei Long, Yuxiang Wang, Shiwei Geng, Xuening Su, Yang Jiao, Quanjia Chen, Yanying Qu

**Affiliations:** Engineering Research Centre of Cotton, Ministry of Education/College of Agriculture, Xinjiang Agricultural University, 311 Nongda East Road, Urumqi, 830052 China

**Keywords:** Cotton, DUF668 gene family, Bioinformatics analysis, Adverse stress, Expression analysis

## Abstract

**Background:**

Domain of unknown function 668 (DUF668) may play a crucial role in the plant growth and developmental response to adverse stress. However, our knowledge of the function of the DUF668 gene family is limited.

**Results:**

Our study was conducted based on the DUF668 gene family identified from cotton genome sequencing. Phylogenetic analysis showed that the DUF668 family genes can be classified into four subgroups in cotton. We identified 32 DUF668 genes, which are distributed on 17 chromosomes and most of them located in the nucleus of *Gossypium hirsutum.* Gene structure and motif analyses revealed that the members of the DUF668 gene family can be clustered in *G. hirsutum* into two broad groups, which are relatively evolutionarily conserved. Transcriptome data analysis showed that the GhDUF668 genes are differentially expressed in different tissues under various stresses (cold, heat, drought, salt, and *Verticillium dahliae*), and expression is generally increased in roots and stems. Promoter and expression analyses indicated that *Gh_DUF668–05, Gh_DUF668–08, Gh_DUF668–11, Gh_DUF668–23* and *Gh_DUF668–28* in *G. hirsutum* might have evolved resistance to adverse stress. Additionally, qRT-PCR revealed that these 5 genes in four cotton lines, KK1543 (drought resistant), Xinluzao 26 (drought sensitive), Zhongzhimian 2 (disease resistant) and Simian 3 (susceptible), under drought and Verticillium wilt stress were all significantly induced. Roots had the highest expression of these 5 genes before and after the treatment. Among them, the expression levels of *Gh_DUF668–08* and *Gh_DUF668–23* increased sharply at 6 h and reached a maximum at 12 h under biotic and abiotic stress, which showed that they might be involved in the process of adverse stress resistance in cotton.

**Conclusion:**

The significant changes in *GhDUF668* expression in the roots after adverse stress indicate that *GhDUF668* is likely to increase plant resistance to stress. This study provides an important theoretical basis for further research on the function of the DUF668 gene family and the molecular mechanism of adverse stress resistance in cotton.

**Supplementary Information:**

The online version contains supplementary material available at 10.1186/s12864-021-07716-w.

## Background

Plant biologists have always been attracted to the structure, function, and evolutionary model of gene families. The interaction and adaptation between the environment and plants are well studied based on the information of these gene families [1]. Among them, the domain of unknown function (DUF) family refers to a certain protein family with unknown functions, and they play a key role in the plant response to stress [2]. In recent years, a large number of species’ genomes have been sequenced, and the number of DUF superfamilies has increased rapidly. As of 2010, the entire family has expanded to DUF2607 [3]. The Pfam database (version 33.1) now includes 18,259 gene families, of which nearly 31% (5645) are composed of DUF families [4]. The rapid development of genomics and proteomics provides important bioinformatics data for the systematic study of DUF superfamily proteins and lays the foundation for the study of these DUF family genes in regulating plant growth and development and responding to biotic and abiotic stresses.

However, there have been some reports of other DUF gene families in many plants. These include the DUF221, DUF810, DUF866, DUF936 and DUF1618 gene families in rice and the DUF581 and DUF724 gene families in Arabidopsis [5–11]. DUF27 confers the ability to bind to ADP-ribose specifically [12]. The DUF283 domain is required for siRNA processing in gene silencing [13, 14], and the DUF538 superfamily has the ability to hydrolyze chlorophyll [15, 16]. A previous study in Arabidopsis showed that ESK1 (AT3g55990) of the DUF231 gene family is a new negative regulator of cold acclimation [17]. Another study showed that it inhibits the expression of ATRDUF1 and ATRDUF2 (both are RING-DUF1117 E3 ubiquitin ligases) [18]. Abscisic acid (ABA) mediates the response to drought stress. The DUF1644 gene OsSIDP366 positively regulates the response to drought and salt stress in rice [19]. Transgenic rice overexpressing OsSIDP366 shows stronger drought resistance and salt tolerance [20]. Other DUF genes have also been characterized to be related to abiotic stresses, and SIDP361 (DUF1644), OsDSR2 (DUF966) and OsDUF810 [6, 7, 19] from the DUF2275 family are regulated by nutritional status and dehydration during development. Overexpression of the salt-inducing gene TaSRHP (containing the DUF581 domain) in wild-type Arabidopsis can enhance its resistance to salt and drought stress [21]. The function of DUF stress tolerance is currently reported in only model plants, while comprehensive DUF gene family analysis in other plant species is rarely reported.

Although some members of the DUF gene family have been identified, a great number of DUF members are still unknown, especially in cotton. The DUF668 family was identified as a conserved domain containing 29 amino acids. However, limited research has been conducted on this gene family. To date, the DUF668 gene family has been reported in only rice [2]. Previous studies have shown that all tetraploid cottons are directly evolved by doubling the genome after crossing the A and D genomes. Among them, the *G. arboreum* (A2-genome) is used as the donor of A genome, and the *G. hirsutum* (D5-genome) is used as the donor of D genome [22–25]. At present, all of the major cotton areas worldwide are threatened by varying degrees of salt, alkali, drought, cold damage and disease [26–30]. It has become an important scientific issue to continuously identify and screen genes with multiple stress resistance functions and develop related molecular markers in cotton research. Genome sequencing has achieved remarkable results in cotton [22–25], making it possible to systematically identify and study gene families in cotton. DUF668 family genes have shown the potential importance of participating in stress resistance in plants [2]. The evolution, function and classification of this gene family in cotton have not been systematically studied. In this study, members of the DUF668 family were systematically identified, and bioinformatic analyses were performed based on cotton genome data. Chromosome distribution, gene replication, promoter cis-acting elements, and expression profiles of the GhDUF668 gene were analyzed in different tissues and under various stresses. qRT-PCR was used to analyze the expression of candidate genes under drought and *Verticillium dahliae* (V991) treatments, revealing their possible biological functions. The results will further broaden our understanding of the roles of DUF668 genes in plants, providing a basis for further research on the functions of these genes in cotton under adverse stresses and laying a foundation for the subsequent analysis of their functions.

## Results

### Identification of the DUF668 gene family from cotton

To investigate the copy number variation in the DUF668 genes during cotton evolution, a comprehensive search was conducted for DUF668 genes across cotton lineages, including *G. arboreum, G. raimondii, G. hirsutum* and *G. barbadense*. The results were verified in the NCBI-CDD database (Figure S1). In the end, there were 17, 17, 32, and 33 sequences in *G. arboreum, G. raimondii, G. hirsutum and G. barbadense*, respectively (Table S1). The results showed that the numbers of DUF668 genes in *G. arboreum* and *G. raimondii* were almost similar as were those in *G. hirsutum* and *G. barbadense*. The DUF668 family genes in two diploid cotton species are basically half of the number in two tetraploid cotton species, which conforms to the known evolutionary relationship of cotton [24, 25], indicating that the DUF668 family is conserved in the evolution of cotton. *Gh_DUF668–01* ~ *Gh_DUF668–32* were named according to the position of the 32 sequences on the chromosome (Table 1) in *G. hirsutum*. The open reading frame (ORF) of the DUF668 family genes in *G. hirsutum* is 630 ~ 1959 bp in length, and the encoded protein contains 209 ~ 652 amino acid residues. The relative molecular mass is between 23.46 and 72.69 kDa, and the theoretical isoelectric point is between 5.29 and 9.83. Each of the family members contains a DUF668 domain. The subcellular localization of proteins showed that 27 were located in the nucleus, 4 were located in the chloroplast, and 1 was located in the inner membrane.

**Table 1:** Information on the DUF668 gene family in *G. hirsutum*

Gene name	Gene ID	Open reading frame/bp	Protein length/aa	Relative molecular weight (r)/kDa	Theoretical isoelectric point (pI)	Subcellular localization
GhDUF668–01	GH_A01G1304	1875	598	67.11	9.28	nucleus
GhDUF668–02	GH_A01G2268	1707	568	64.86	9.22	nucleus
GhDUF668–03	GH_A01G2392	1392	463	52.72	9.2	chloroplast
GhDUF668–04	GH_A02G0391	1788	595	67.2	6.95	nucleus
GhDUF668–05	GH_A02G0661	1959	652	72.62	9.45	nucleus
GhDUF668–06	GH_A02G0964	1113	370	41.94	9.72	nucleus
GhDUF668–07	GH_A04G1034	1539	512	58.73	9.36	nucleus
GhDUF668–08	GH_A05G1859	1854	617	68.53	9.49	nucleus
GhDUF668–09	GH_A05G2503	1947	648	71.88	9.29	nucleus
GhDUF668–10	GH_A05G4150	1758	585	65.7	9.16	nucleus
GhDUF668–11	GH_A07G2347	1875	624	69.82	8.99	nucleus
GhDUF668–12	GH_A09G0607	1353	450	51.07	9.23	nucleus
GhDUF668–13	GH_A09G1530	1617	538	61.45	9.83	nucleus
GhDUF668–14	GH_A09G2457	1782	593	66.63	8.31	nucleus
GhDUF668–15	GH_A11G0076	1386	461	51.95	9.43	chloroplast
GhDUF668–16	GH_A12G2838	1179	392	44.97	9.24	chloroplast
GhDUF668–17	GH_A13G1138	1455	484	54.28	8.96	nucleus
GhDUF668–18	GH_D01G1376	1797	598	67.05	9.2	nucleus
GhDUF668–19	GH_D01G2352	1707	568	64.73	9.27	nucleus
GhDUF668–20	GH_D01G2470	1392	463	52.66	9.01	chloroplast
GhDUF668–21	GH_D02G0412	1791	596	67.2	7.55	nucleus
GhDUF668–22	GH_D02G0670	1959	652	72.69	9.43	nucleus
GhDUF668–23	GH_D02G1007	1836	611	68.02	9.59	nucleus
GhDUF668–24	GH_D04G0230	630	209	23.46	5.29	nucleus
GhDUF668–25	GH_D04G1367	1551	516	59.29	9.36	nucleus
GhDUF668–26	GH_D05G1897	1854	617	68.75	9.56	nucleus
GhDUF668–27	GH_D05G2525	1947	648	71.93	9.16	nucleus
GhDUF668–28	GH_D07G2291	1875	624	69.64	9.06	nucleus
GhDUF668–29	GH_D09G0545	1356	451	51.18	9.27	nucleus
GhDUF668–30	GH_D09G1537	1617	538	61.58	9.79	nucleus
GhDUF668–31	GH_D11G0081	1386	461	52.1	9.37	chloroplast
GhDUF668–32	GH_D12G2861	852	283	32.24	9.19	endomembrane

**Fig. 1:**
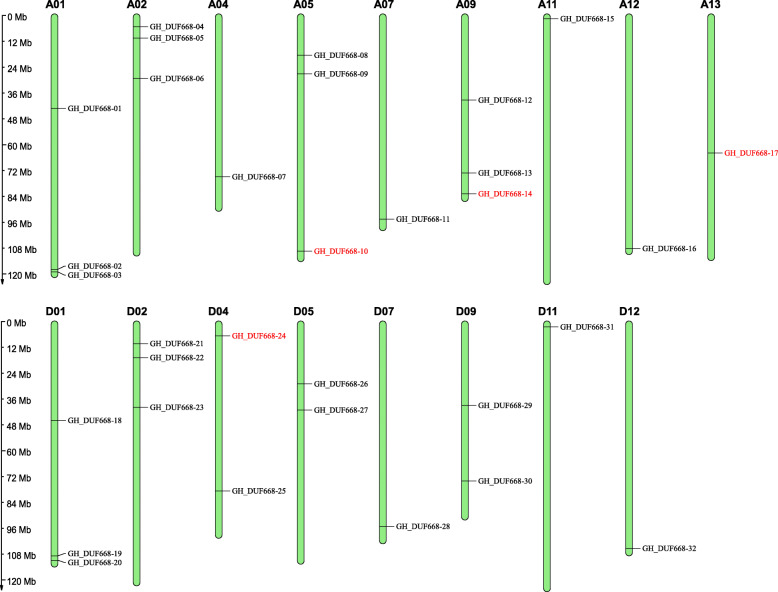
Chromosome locations of the *G. hirsutum* DUF668 genes. The gene name with red color indicates that there is no homologous gene at the corresponding position on its corresponding chromosome

Thirty-two GhDUF668 genes were distributed on 17 chromosomes (A01, A02, A04, A05, A07, A09, A11, A12, A13, D01, D02, D04, D05, D07, D09, D11, and D12) of *G. hirsutum* (Fig. 1). Subgroup A and subgroup D contained 17 and 15 sequences, respectively. Previous studies suggested that *G. arboreum* and *G. raimondii* were donor species for subgenome A and subgenome D, respectively. The number of GhDUF668 genes in subgenome A was consistent with the number of GaDUF668 genes, and two of the DUF668 genes were missing from subgenome D compared to the number of GrDUF668 genes. This result indicated that subgroup D might have lost genes due to redundant gene functions during cotton evolution. Only one sequence of this family was on chromosome A04, while chromosome D04 in *G. hirsutum* contained two sequences. Three sequences were observed on chromosomes A05 and A09, while chromosomes D05 and D09 contained two sequences. The A13 chromosome contained one sequence, but the GhDUF668 gene sequence was not contained in D13 chromosome. This result showed that the DUF668 genes might have been lost and duplicated in the process of evolution. However, there was a strong correlation between subgroup A and subgroup D, which was also in line with the evolutionary relationship in cotton [22–25].

### Phylogenetic analysis of the DUF668 gene family in cotton

**Fig. 2:**
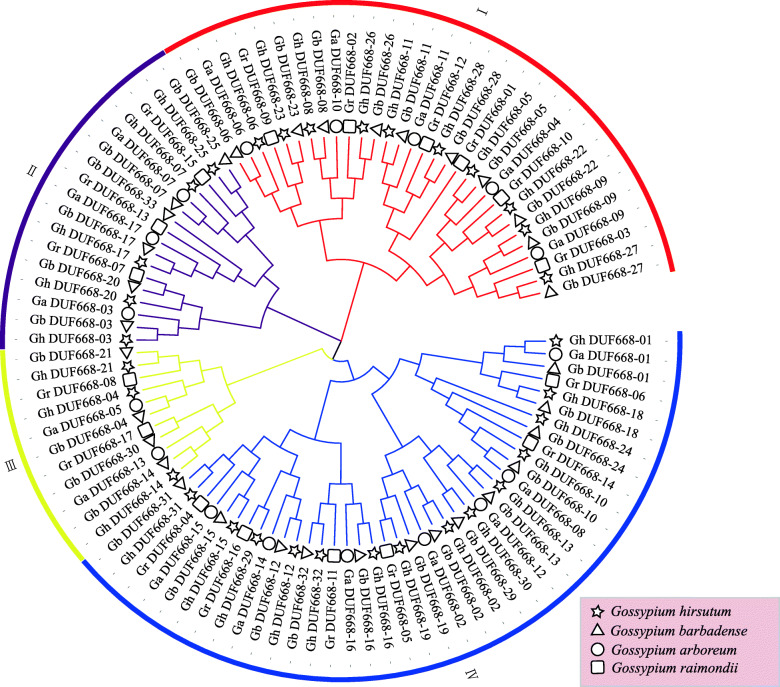
Phylogenic tree of the DUF668 family members in *G. arboreum*, *G. raimondii*, *G. hirsutum* and *G. barbadense*

To explore the phylogenetic relationship of the cotton DUF668 genes, a phylogenetic tree was constructed. DUF668 gene protein sequences (Table S2) from four different cotton subspecies were used. All of the DUF668 proteins can be divided into 4 subgroups (Fig. 2). The number of DUF668 genes in each subgroup of *G. hirsutum* and *G. barbadense* was basically twice the number in each subgroup of *G. arboreum* and *G. raimondii*. This was consistent with the results of the previous analysis and conforms to the evolutionary relationship in cotton. The results showed that the DUF668 genes were relatively conserved in evolution in cotton. Although the third subgroup had relatively few members, they were retained during evolution in cotton [22–25], which indicated that they may play an important role in biological processes.

**Fig. 3:**
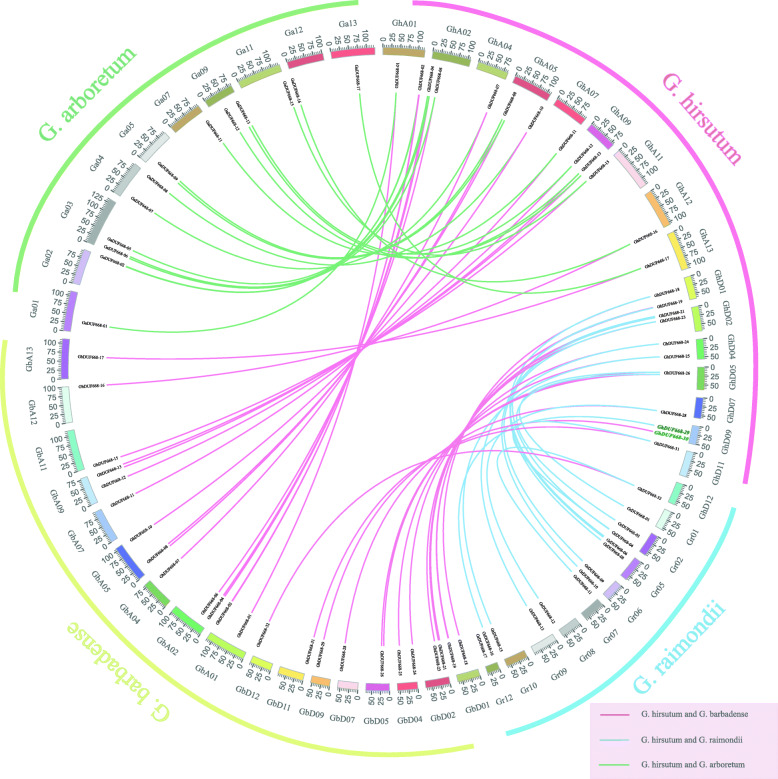
Collinearity analysis of DUF668 family members in *G. arboreum, G. raimondii, G. hirsutum* and *G. barbadense.* The green line represents the collinearity of DUF668 gene from subgenome A in *G. hirsutum*, and *G. arboreum* . The red line represents the collinearity of DUF668 gene in *G. hirsutum*, and *G. barbadense*. Blue line represents the collinearity of DUF668 gene from subgenome D in *G. hirsutum*, and G. raimondii

According to the number of genes, chromosome location and phylogenetic tree analysis, DUF668 was predicted to be relatively conserved in cotton. To study the evolutionary relationship of DUF668, we selected *G. hirsutum* as the core and constructed the collinearity relationship in *G. hirsutum* related to other cotton species (Fig. 3). We found that 13 sequences for DUF668 family genes from the subgenome A in *G. hirsutum* had collinearity with 17 sequences in *G. arboreum* and *G. barbadense*. Except for the *Gh_DUF668–30* gene, one sequence for the DUF668 family genes in the subgenome D in *G. hirsutum* had collinearity with one sequence in *G. raimondii* and *G. hirsutum*. However, 11 sequences in *G. barbadense* and 13 sequences in *G. raimondii* had collinearity with 15 and 14 sequences in *G. hirsutum,* respectively. This was basically consistent with the analytical results of the DUF668 family genes in the A subgroup. Surprisingly, except for *Gh_DUF668–29* and *Gh_DUF668–30*, each sequence of DUF668 family genes in either subgenome A or D in *G. hirsutum* corresponded to only one sequence in *G. arboreum* and *G. barbadense*. This shows that the DUF668 family genes may have been lost during evolution in *G. hirsutum*; later, they were duplicated due to functional requirements, making them consistent with the number in *G. arboreum*. This illustrated the complexity of DUF668 family gene functions.

In order to further study the evolutionary relationship of DUG668 gene family in cotton. Protein sequences of duf668 gene family from *Arabidopsis thaliana*, rice and four different cotton subspecies were selected to construct an evolutionary tree and 10 different conserved motifs (Figure S2) were identified [2]. The evolutionary tree showed that it could be divided into four categories, which was consistent with cotton. Motif 3, 5.6.7.10 are the most common, which are found in all sequences. Motif 1 and 9 are specific elements of the fourth branch, motif 2 and 4 are specific sequences in addition to the fourth branch. In conclusion, the motifs of DUF668 gene are consistent with their phylogenetic relationships. This indicates that DUF668 gene family has internal differentiation in the process of evolution, which may further lead to functional differentiation.

### Phylogenic tree, motif and gene structure of the DUF6688 genes in *G. hirsutum*

**Fig. 4:**
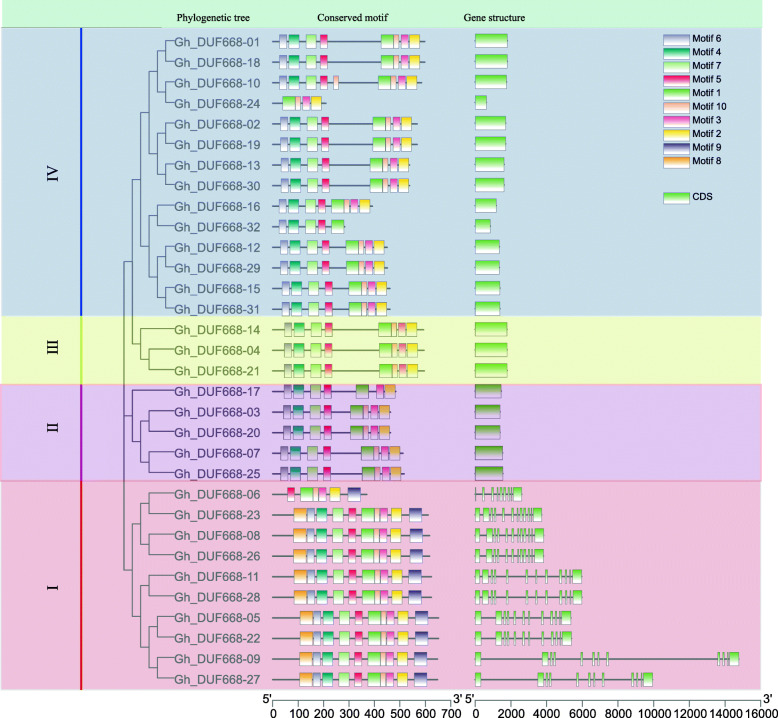
Phylogenic tree, motif and gene structure of GhDUF668 genes in *G. hirsutum.* I, II, III and IV are grouped according to the result of phylogenic tree

The phylogenetic tree, gene structure and motif were analyzed according to the full-length coding sequence (CDS) and protein sequence of the GhDUF668 genes (Fig. 4, Fig. S3). Except for *GhDUF668–06, 24* and *32*, the rest of the members had the same motif (*1, 2, 3, 4, 5, 6, 7, 10)*, indicating that the same family members had similar functions. Besides the first subgroup, one exon and four identical motifs (*1, 2, 3, 10*) were observed in other subgroups, whereas introns were not contained. However, the length between the exons was different. Except for *GhDUF668–06,* which contained 6 motifs (*1, 2, 3, 5, 9, 10*) and 6 exons, the first broad group contained 10 motifs and 12 exons. The difference between the structures of *GhDUF668–06*, *24* and *32* in the same group might be due to changes in the function of the gene or errors in genome annotation. Further study is required. A motif is a structural component with a specific spatial conformation and function in a protein molecule, which is a subunit of a structural domain and connects with a specific function. This result suggests that the first broad group might have changed its gene structure during the evolutionary process and might have a more important function in cotton growth and development than originally thought.

### Cis-acting element analysis of the DUF668 gene in *G. hirsutum*

**Fig. 5:**
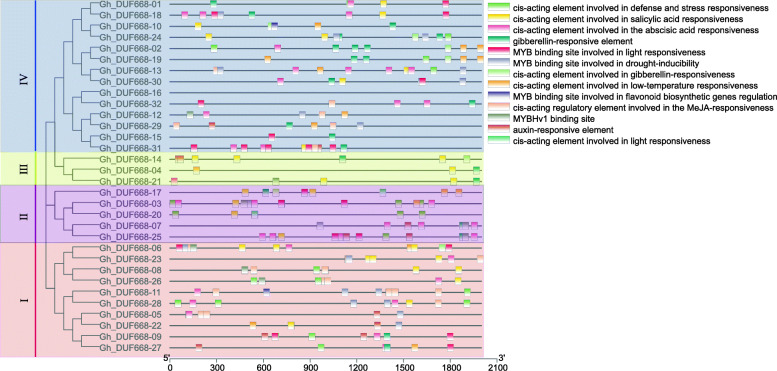
Cis-acting element analysis of DUF668 family members in *G. hirsutum.* I, II, III and IV are grouped according to the result of phylogenic tree

The 2000 bp promoter region upstream of the GhDUF668 genes was extensively analyzed. Various cis-acting elements were found in defense mechanisms, stress responses, salicylic acid, ABA, gibberellin, auxin, jasmonic acid, light responses, drought induction, MYB binding sites for flavonoid synthesis, and responses to low temperature, which are related to plant hormones and environmental stress (Fig. 5, Table S3). Previous grouping results showed that the first group contained more cis-acting elements than the other groups, indicating that the first subgroup might have a more important function under adverse stress conditions in cotton growth and development. Each GhDUF668 promoter contained different numbers and types of cis-acting elements, indicating that they might participate in different biotic and abiotic stress responses through the different signaling pathways.

### Tissue-specific expression analysis of DUF668 genes in *G. hirsutum*

**Fig. 6:**
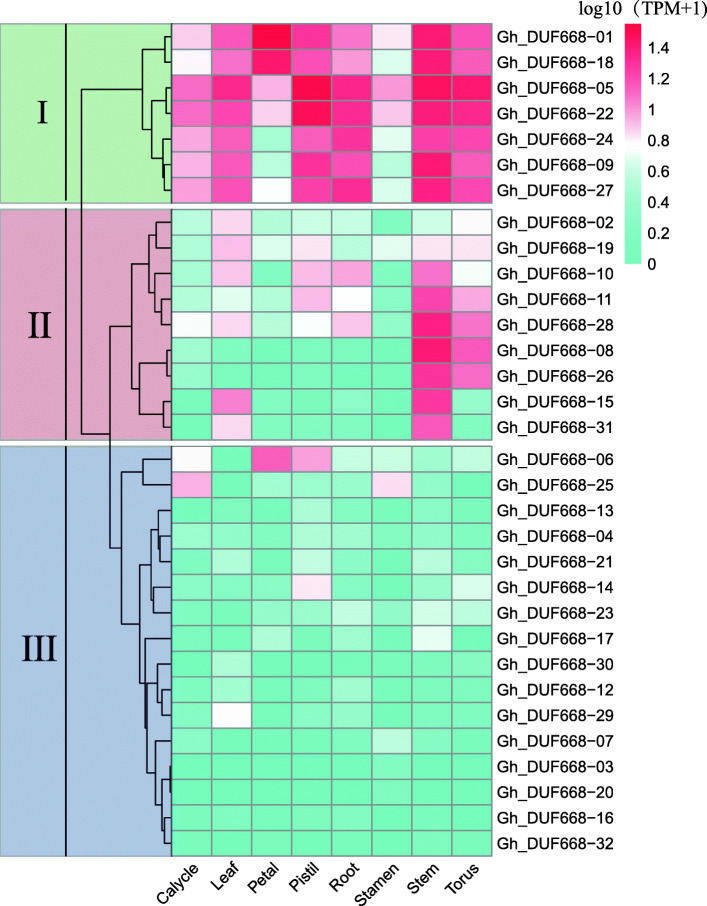
Tissue-specific expression analysis of DUF668 genes from *G. hirsutum* based on transcriptome data. I, II and III are grouped and clustered according to the expression pattern

Gene expression patterns are usually related to gene functions. Our analysis of the expression patterns of the GhDUF668 gene in roots, stems, leaves, pistils, stamens, calyxes, petals and receptacles in cotton showed that most of the selected 32 GhDUF668 genes had tissue expression specificity (Fig. 6) that can be divided into 3 expression patterns. Seven genes (*GhDUF668–01, 18, 05, 22, 24, 09, 27*) could be divided into the first expression pattern, and expression was mainly expressed in the pistil, in roots, stems, and receptacles with the lowest expression in petals and stamens. Nine genes (*GhDUF668–02, 08, 10, 11, 15, 19, 26, 28, 31*) could be divided into the second expression pattern, With most of them expressed in the stem. The rest were classified as the third expression pattern, and the expression level of the eight tissues in this pattern was low. The gene expression of the GhDUF668 genes was specific and contained more complex functions.

### Expression analysis of the DUF668 gene in *G. hirsutum* in response to stress

**Fig. 7:**
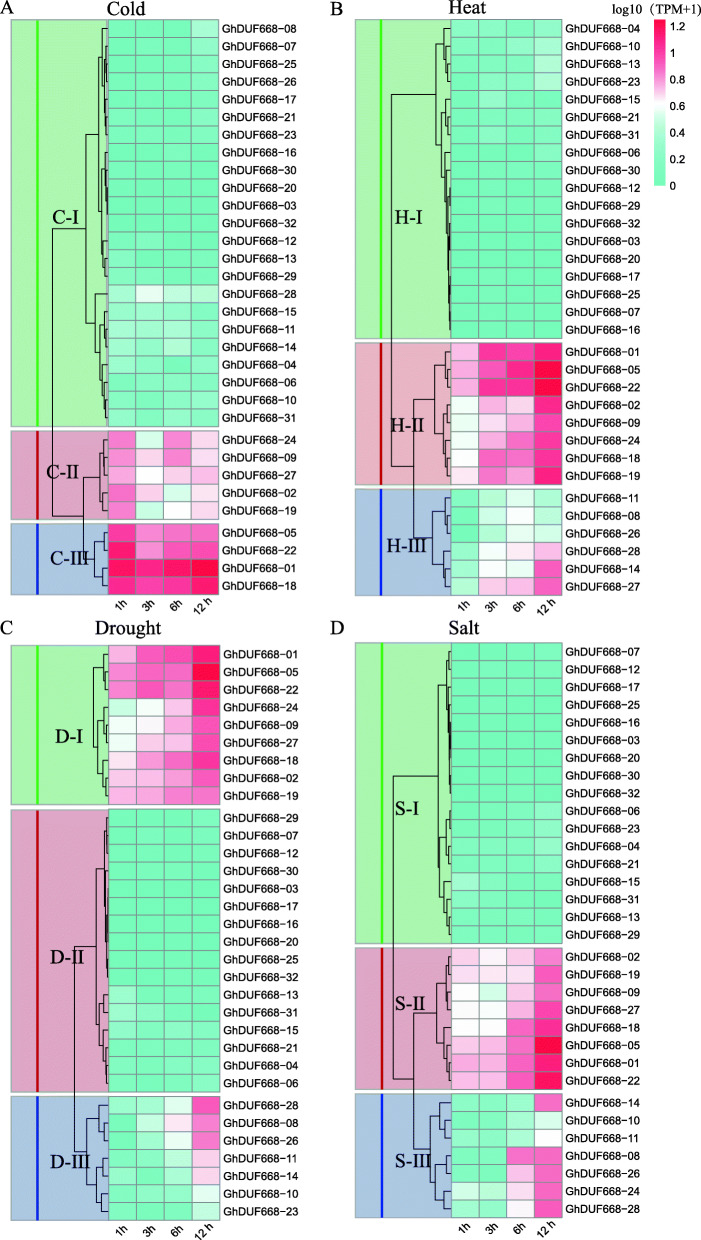
Expression analysis of the DUF668 genes from *G. hirsutum* under cold (**a**), heat (**b**), drought (**c**) and salt (**d**) stress based on transcriptome data. I, II and III are grouped and clustered according to the expression pattern. C, H, D, S represent cold, heat, drought and salt, respectively

The expression analysis of the GhDUF668 genes after cold, heat, drought and salt treatment showed that the expression patterns of the GhDUF668 genes can be divided into three categories (Fig. 7). After cold treatment, the expression of nine genes (*GhDUF668–****01, − 02, − 05, − 09, − 18, − 19, − 22, − 24, − 27***) from the C-II and C-III categories significantly changed (Fig. 7a), indicating that the expression of these nine genes could be induced by cold stress. These genes might play a corresponding role in the response to cold stress in *G. hirsutum*. Under heat stress conditions, the expression of 14 genes (*GhDUF668–****01, − 02, − 05,*** *− 08, −* ***09,*** *− 11, − 14, −* ***18, − 19, − 22, − 24,*** *− 26, −* ***27,*** *− 28*) from the H-II and H-III categories was obviously upregulated and reached the maximum value at 12 h (h) (Fig. 7b), indicating that these genes might play a role in heat resistance in *G. hirsutum*. Polyethylene glycol (PEG) was used to simulate drought stress. The expression of 16 genes (*GhDUF668–****01, − 02, − 05,*** *− 08, −* ***09,*** *− 10, − 11, − 14, −* ***18, − 19,*** *−* ***22,*** *− 23, −* ***24,*** *− 26, −* ***27,*** *− 28*) was upregulated and reached a maximum at 12 h (Fig. 7c), indicating that these 16 genes might play a role in drought resistance in cotton. After salt stress treatment, the expression of 15 genes (*GhDUF668–****01, − 02, − 05,*** *− 08,-* ***09,*** *− 10, − 11, − 14, −* ***18, − 19, − 22, − 24,*** *− 26,* ***− 27,*** *− 28*) from the S-II and S-III categories was upregulated and reached a maximum at 12 h (Fig. 7d), indicating that these 15 genes might play a role in salt tolerance in cotton. In summary, nine genes (*GhDUF668–****01, − 02, − 05, − 09, − 18, − 19, − 22, − 24, − 27***) were induced by various abiotic stresses, implying that they might be affected by nonbiological stress in cotton. Although most of the genes had obvious expression changes and reached their maximum at 12 h, RNA-seq measurement was performed at only 12 h. We speculated that these genes might continue to increase their expression after 12 h under abiotic stress conditions *in G. hirsutum*. In summary, the expression levels of approximately half of the GhDUF668 genes significantly changed with higher tissue-specific expression levels under abiotic stress conditions. This result indicated that the GhDUF668 genes might play a role in the abiotic stress response of *G. hirsutum.* However, further verification is needed.

### Expression analysis of the DUF668 gene in *G. hirsutum* in response to Verticillium wilt stress

**Fig. 8:**
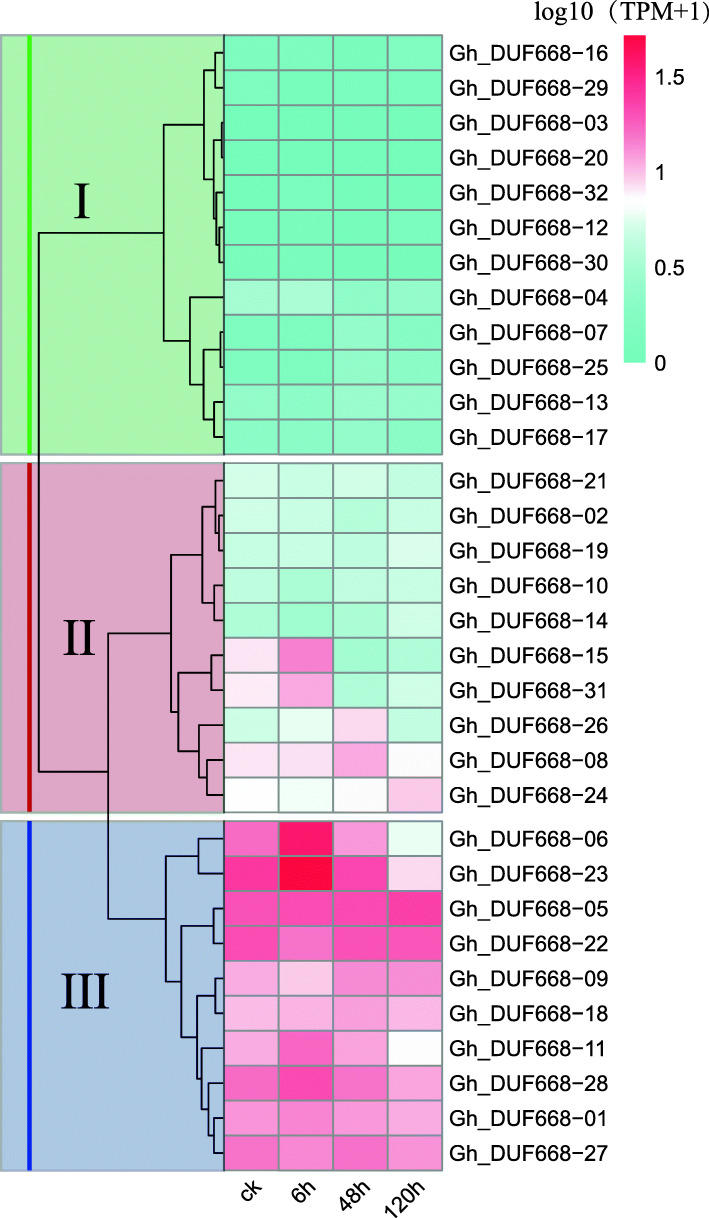
Expression analysis of the DUF668 genes from *G. hirsutum* under *V. dahliae* stress based on transcriptome data. I, II and III are grouped and clustered according to the expression pattern

Cotton production is restricted by *V. dahliae*, which has a serious impact and causes great economic losses every year. This study was based on the transcriptomic data of cotton roots after inoculation with *V. dahliae*. The results showed (Fig. 8) that these GhDUF668 genes could be clustered into three expression patterns within 0 ~ 120 h. The expression of 20 genes (*GhDUF688–01, − 02, − 05, − 06, − 08, − 09, − 10, − 11, − 14, − 15, − 18, − 19, − 21, − 22, − 23, − 24, − 26, − 27, − 28* and − *31*) from the second and third expression patterns changed. Among them, the expression levels of six genes (*GhDUF688–06, − 11, − 15, − 23, − 28,* and − *31*) reached a maximum at 6 h and then continued to decline, indicating that these six genes might be involved in the response to *V. dahliae* treatment in the early stage of cotton. The expression levels of *GhDUF688–08* and *GhDUF688–26* reached a maximum at 48 h and then decreased, indicating that these two genes may play a role in the middle and late stages in response to *V. dahliae* infection in cotton.

### Response of the DUF668 gene in *G. hirsutum* to drought and Verticillium wilt stress by qRT-PCR

**Fig. 9:**
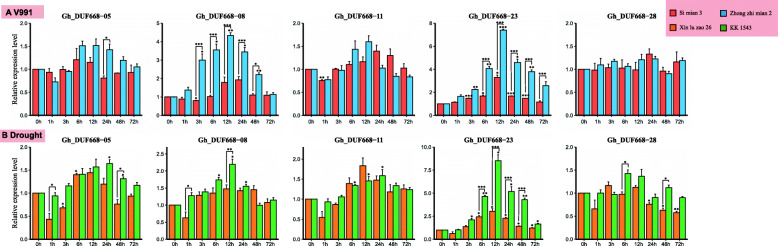
Expression profiling of the GhDUF668 genes in root tissue under drought and Verticillium wilt conditions. The error bars represent the means of three technical replicates ± SEs. The X-axis represents the treatment time, and the Y-axis represents the relative expression of genes。Statistically significant differences from the control group are indicated as *P < 0.1; **P < 0.05; ***P < 0.01

Adverse stresses can cause transcriptome reprogramming events. Our previous analysis showed that most of the expression of the GhDUF668 genes undergoes significant changes under stress treatment conditions. Based on the transcriptome expression profiles and promoter cis-acting elements, we speculated that five genes (*GhDUF668–05, − 08, − 11, − 23* and *− 28*) from the GhDUF668 genes might be involved in resistance to stress conditions. We selected KK 1543 (drought-resistant), Xinluzao 26 (drought-susceptible), Zhongzhimian 2 (disease-resistant) [28] and Simian 3 (disease-susceptible) (unpublished) to determine whether these genes were involved in the response to adverse stress. qRT-PCR was used to detect the transcription levels of these five GhDUF668 genes in roots under drought and Verticillium wilt treatment at the seedling stage. Compared with that at 0 h, the expression of these 5 genes was significantly different after the roots were stressed (Fig. 9). The transcription levels of these five genes were significantly induced after adverse stress treatment.

After the roots of disease-resistant and susceptible materials were inoculated with *V. dahlia*, the RNA transcription levels of selected genes were significantly induced at different periods in the two materials, while except for Gh-DUF668–28. Thus, the expression levels were significantly increased, suggesting that these genes might participate in the process of responding to the invasion of V991 in cotton. Among them, *GhDUF668–08* and *GhDUF668–23* reached their maximum values at 12 h in disease-resistant materials. The maximum expression of GhDUF668–08 was observed at 24 h in susceptible materials, and expression was lower in the susceptible materials than in the disease-resistant materials. Although *GhDUF668–08* and *GhDUF668–23* were significantly induced in both materials, the expression level in disease-resistant materials was dramatically higher than that in susceptible materials. Minor changes in the expression levels of the other three genes were observed. In summary, these five genes might play a role in the process of responding to the invasion of *V. dahlia* in cotton. Among them, *GhDUF668–08* and *GhDUF668–23* might play leading roles in disease resistance.

After PEG simulated drought treatment, the transcription levels of all selected genes were significantly induced at different periods in the two materials, and the expression levels were significantly increased, suggesting that these genes may be involved in the response to drought conditions in *G. hirsutum*. *GhDUF668–08* and *GhDUF668–23* were significantly induced at 6 h, and their expression increased sharply, reaching a maximum at 12 h. The changing expression trends of *GhDUF668–08* and *GhDUF668–23* under drought treatment were consistent with those of inoculation with V991. However, the expression level of *GhDUF668–08* in *G. hirsutum* under drought treatment was not significantly different from that in *G. hirsutum* inoculated with *V. dahlia*. In summary, these 5 genes might have a role in the drought stress response in cotton. Furthermore, *GhDUF668–23* might play a leading role in this process.

The same expression pattern was observed among five genes (*GhDUF668–05, − 08, − 11, − 23* and *− 28*) in different materials under biotic stress and abiotic stress. Compared with expression at 0 h, expression of 5 genes at other timepoints changed significantly. This implied that the expression of these genes was regulated by adverse stresses and might play a certain role in the process of responding to adverse stress in *G. hirsutum*. Among them, the expression levels of both *GhDUF668–08* and *GhDUF668–23* increased sharply at 6 h and reached a maximum at 12 h with drought stress and *V. dahlia* inoculation treatment, respectively. The expression levels of *GhDUF668–08* and *GhDUF668–23* were significantly upregulated among the five genes in the same period. This further shows that *GhDUF668–08* and *GhDUF668–23* might play certain roles in the response to biotic and abiotic stress in cotton.

### Tissue-specific expression analysis of the DUF668 gene *in G. hirsutum*

**Fig. 10:**
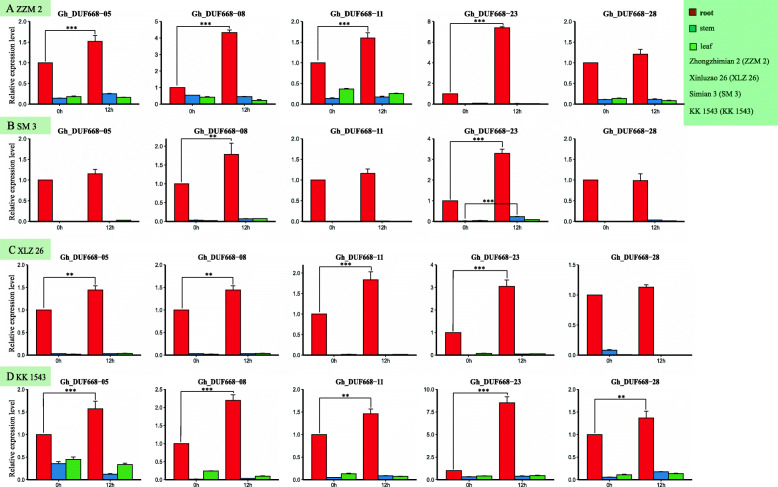
Tissue-specific expression analysis of the GhDUF668 genes under drought and Verticillium wilt stress. The error bars represent the means of three technical replicates ± SEs. The X-axis represents the treatment time, and the Y-axis represents the relative expression of genes. Statistically significant differences from the control group are indicated as *P < 0.1; **P < 0.05; ***P < 0.01. **a** and **b** Images of tissue-specific expressions of Zhongzhimian 2 and Simian 3 under the stress of Verticillium dahliae. **c** and **d** Images of expressions of Xinluzao 26 and KK1543 under drought stress

We proved that the expression of *GhDUF668–08* and *GhDUF668–23* changed dramatically under different stress conditions, and significant differences in their expression were observed among different materials. Moreover, due to the specific parts of gene expression, the molecular biological functions were different. The first part of the plants to sense the response to drought stress and pathogen invasion is the root. For this reason, we selected the periods that could strongly verify the tissue-specific expression of these five candidate genes in the four materials during treatment. We found that the expression in roots was significantly higher than that in stems and leaves (Fig. 10). Interestingly, expression of these five genes changed significantly in the roots before and after different stresses, but severe changes occurred in the stems and leaves. Among them, the *GhDUF668–08* and *GhDUF668–23* genes changed significantly in the roots. This result showed that the increased expression of the GhDUF668 genes in the roots was likely to enhance cotton resistance. These results further illustrated the certain role of the GhDUF668 genes in stress resistance.

### Analysis of protein interaction networks

**Fig. 11:**
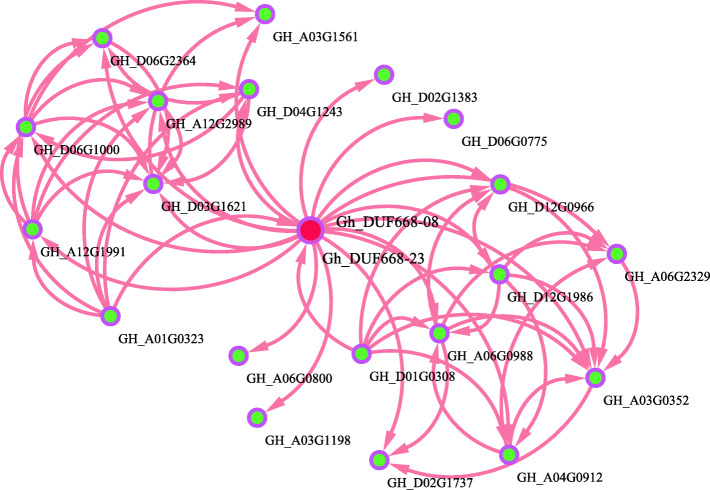
Interaction network of *GhDUF668–08* and *GhDUF668–23* proteins in *G. hirsutum*

As we all know, rarely single protein can directly participate in plant stress response. Most of plant physiological processes are completed by protein-protein interaction. For better understanding the molecular mechanism of *GhDUF668–08* and *GhDUF668–23* genes The interaction network between *GhDUF668–08* and *GhDUF668–23* genes proteins and other *Gossypium hirsutum* proteins was constructed (Fig. 11), based on the interaction network of Arabidopsis homologous genes (Figure S4). The interaction between *GhDUF668–08*, *GhDUF668–23* proteins with 20 *Gossypium hirsutum* proteins was found. Among them, *GH_A03G1561 and GH_D01G0308* has played a potential role in response to stress in *G. hirsutum.* Only *GH_A01G0323* and *GH_D01G0308* are located upstream of *GhDUF668–08* and *GhDUF668–23*, which indicates that *GH_A01G0323*and *GH_D01G0308* may interact with the DUF3475 domain of *GhDUF668–08* and *GhDUF668–23*. However, more genes are combined with the downstream DUF668 domain of *GhDUF668–08* and *GhDUF668–23,* which further indicates the complex function of DUF668 gene family and the potential role of *GhDUF668–08* and *GhDUF668–23* in response to stress in *G. hirsutum*. Our results lay a molecular foundation to further investigate the function and molecular mechanism of these genes in stress tolerance.

## Discussion

Many studies have shown that genome-wide identification and expression analysis could help researchers understand the origin, diversity and biological functions of these DUF gene families [2, 5, 8, 9, 31]. In recent years, with the improvement of sequencing technology and the decrease in sequencing cost, cotton genome sequencing has been continuously improved and updated, laying the foundation for studying gene functions at the comprehensive genome level [22–25]. This study further deepens the knowledge of cotton genomics and genetics, providing the possibility to discover members of the DUF668 gene family in cotton and their phylogenetic relationships. At present, research on cotton DUF668 family genes has not been reported.

Our research shows that more DUF668 family genes exist in cotton than in monocot and dicot plants [2]. This might contribute to the double duplication of the cotton genome, or it might be that more genes were annotated in cotton than in other species [24, 25, 32, 33]. The number of genes, phylogenetic tree and collinearity showed (Figs. 2 and 3) that the cotton DUF668 gene family was conserved during the evolution of cotton, which was consistent with the evolutionary relationships between cotton species [34–37]. In cotton, a multitude of gene family members with high sequence similarity have been observed to cluster on chromosomes as paralogous pairs [38–40]. However, the GhDUF668 genes did not do so, proving that this gene family has not undergone gene amplification events, including tandem and fragment duplication, during evolution. Protein structure and gene structure are closely related to gene function. The numbers of intron/exon might represent splicing variants and were used to classify genes [41, 42]. According to the distribution and quantity of gene structures and motifs analysis, DUF668 members were divided into 2 broad groups in *G. hirsutum*, which was consistent with research in rice [2]. The number and structure of genes in each broad group were significantly different (Fig. 4). Moreover, the number of genes from the two broad groups was basically the same in rice and some Poaceae [2]. Although the second broad group contains the same number of CDS and motif except for Gh_DUF668–06, from the perspective of the full length of genes, the number and length of similar CDS have different gene lengths, indicating that the intron length has a larger difference. Surprisingly, we found that in addition to *GhDUF668–06* and *GhDUF668–24*, the DUF668 gene family contains not only the DUF668 domain but also the DUF3475 domain in *G. arboreum, G. raimondii* and *G. hirsutum.* However, the DUF3475 domain in plants has not been reported. We clearly found that motif is located in the two domains respectively (Fig. 4, Fig. S1). Our results showed that the GhDUF668 genes might belong to the same family as GhDUF3475 in cotton. DUF668 contains more complex functions and may also be related to the DUF3475 domain. Therefore, we speculated that the DUF3475 domain might bind to upstream transcription factors, and certain functions were performed by regulating downstream genes through DUF668. These results indicate that this gene family plays a more important role in the growth and development of cotton.

Expression patterns of genes can provide important clues for characterizing gene function, which is thought to be related to promoter region differentiation. Through the analysis of promoters, tissue-specific expression and expression profiles (Figs. 5, 6, 7, 8), we found that *Gh_DUF668–05, Gh_DUF668–08, Gh_DUF668–11, Gh_DUF668–23* and *Gh_DUF668–28* contained more homeopathic action elements, and higher expression was observed under adverse stress conditions. We speculated that these five genes might participate in resistance to stresses in cotton development. However, we were surprised to find that these five genes all came from the second broad group. Although the second broad group contains a relatively small number of genes, they have been retained during the evolution of cotton, implying that they may play important roles in biological processes. Regardless of whether plants are subjected to biological or abiotic stress, their initial sensing parts are basically at the roots [43–45]. Moreover, analysis of the gene expression in different tissues and the molecular biological functions were performed differentially [46, 47]. The *Gh_DUF668–08* and *Gh_DUF668–23* expression levels both increased sharply at 6 h and reached a maximum at 12 h under biotic and abiotic stresses. An obvious difference was found between these two genetically resistant materials in the same treatment period. Moreover, we found that expression in the roots was significantly higher than that in the stems and leaves. However, minor changes in the stems and leaves were observed. Among the genes, *Gh_DUF668–08* and *Gh_DUF668–23* had the most obvious changes in the roots.

Many studies have shown that salicylic acid (SA), methyl jasmonate (MeJA) and ABA are apparently related to plant resistance [48–52]. A large number of cis-acting elements were contained in *Gh_DUF668–08* and *Gh_DUF668–23*, which are involved in defense mechanisms, responses to stresses, and SA, ABA, MeJA and MYB binding sites (Fig. 5). Homology analysis showed that *Gh_DUF668–08* and *Gh_DUF668–23* have the highest homology with *Arabidopsis AT1G30755*, which is highly related to growth, development and defense in Arabidopsis [53]. In a study conducted with flg22 treatment in Arabidopsis, the expression levels of 35 genes that contain the DUF domain were obviously changed, and DUF668 members were included [54]. The results indicate that *Gh_DUF668–08* and *Gh_DUF668–23* play leading roles in the response to biotic and abiotic stresses in cotton.

Thus, cotton tissues and expression profiles under various stress conditions were analyzed using qRT-PCR to detect the GhDUF668 genes. It was shown that most members of this family were very important in the process of resistance to one or more stresses in *G. hirsutum*. Moreover, we speculated that the apparent changes in GhDUF668 gene expression in the roots were modulated by adverse stresses, which caused the enhancement of cotton resistance. These results laid the foundation for us to further verify its function and the molecular mechanism of stress resistance.

## Conclusions

In this study, cotton DUF668 genes were identified in the whole genome. According to the number of genes and phylogenetic tree and collinearity analyses, the cotton DUF668 family genes could be divided into 4 subgroups. Thirty-two DUF668 genes were observed in the *G. hirsutum* genome, and their structural characteristics and expression patterns were analyzed. We found that the genes from the GhDUF668 gene family could be divided into two broad groups. *GhDUF668–08* and *GhDUF668–23*, which might play leading roles in the response of biotic and abiotic stress in cotton. This study groundbreakingly conducted a systematic analysis with the cotton DUF668 gene family and provided a new understanding of cotton resistance to stress, laying the foundation for the in-depth functional analysis and breeding application of these genes.

## Methods

### Plant material

Based on the previous research results of our research team, expression analysis of KK1543 (drought resistance, Xinjiang Bazhou Agricultural Institute), Xinluzao 26 (drought susceptibility, Xinjiang Academy of Agricultural Sciences), Zhongzhimian 2 (disease resistance, The Institute of Plant Protection, Chinese Academy of Agricultural Sciences) and Simian 3 (disease susceptibility, Jiangsu Academy of Agricultural Sciences) materials was conducted under drought and Verticillium wilt (V991) inoculation. The seeds of KK1543 and Xinluzao 26 were germinated at 28 °C under a 16 h light/8 h dark cycle and then transplanted into normal hydroponic solution. Hoagland nutrient solution was applied every 2 days. KK1543 and Xinluzao 26 were subjected to drought treatment with 15% PEG6000 at the two-leaf stage. Zhongzhimian 2 and Si Mian 3 were planted in soil pots [28]. The middle and lower parts of the pot were cut with a small knife in the two-leaf stage to ensure that the roots were damaged, then *V. dahliae* 991, the spore suspension (1 × 10^7^ spores Ml/L) was watered along the roots of cotton seedlings to ensure they were successfully inoculated. The root tissues of the two treatments were collected at 0, 1, 3, 6, 12, 24, 48, and 72 h.

### Identification and bioinformatics analysis of the cotton DUF668 gene

This study used the latest published genome sequence as a reference. The relevant genomic and proteomic data in *G. arboreum, G. raimondii*, *G. hirsutum,* and *G. barbadense* were downloaded from COTTONGEN (http://www.cottongen.org/) [55]. Using the hidden Markov model (PF05003) of the DUF668 gene domain, the protein sequence was identified in cotton through the hidden Markov model (HMM) from HMMER3.2.1 software (http://hmmer.org/) [4, 56]. After the redundancies were removed, domain verification for candidate genes was conducted in the NCBI-CDD (https://www.ncbi.nlm.nih.gov/cdd/) database. Therefore, cotton DUF668 gene family members were further confirmed [57]. The number of amino acid residues, relative molecular mass, and theoretical isoelectric point of the GhDUF668 protein were calculated by ExPASy software (http://cn.expasy.org/tools) [58]. Subcellular localization of the GhDUF668 protein was predicted by EuLoc online software (http://euloc.mbc.nctu.edu.tw/) [59].

### Phylogeny and collinearity analysis of the cotton DUF668 gene family

DUF668 protein sequences identified from *G. arboreum, G. raimondii*, *G. hirsutum,* and *G. barbadense* were subjected to alignment by Clustal W with the default settings in MEGA 7 software. A phylogenetic tree was constructed based on the results of the sequence alignment by using neighbor-joining (NJ), and the bootstrap value setting was 1000 [60]. The phylogenetic tree was edited and displayed with the online tool Evolview (https://evolgenius.info/) [61]. The chromosomal positions and collinearity of the DUF668 genes in cotton were visualized through Circos (http://circos.ca/).

### Chromosome location, gene structure and motif analysis of the DUF668 gene family in *G. hirsutum*

The chromosome location information of DUF668 gene family members was extracted from the *G. hirsutum* genome annotation file, and the chromosome location map of the GhDUF668 gene was redrawn with Mapchart software [62]. The phylogenetic tree of the GhDUF668 genes was constructed, and the nwk file was obtained through MEGA7 software. Motif analysis was performed using the MEME program (the maximum number of motifs was set to 10, [63]. The xml file obtained after running the MEME program, the nwk file obtained from the evolutionary tree, and the gff file obtained from the gene structure were processed and visualized with TBtools software [64].

### Analysis of upstream cis-acting elements of the DUF668 gene

The upstream DNA sequences (2000 bp) of the GhDUF668 genes were obtained. Then, the possible cis-acting elements were predicted through the PlantCARE database (http://bioinformatics.psb.ugent.be/webtools/plantcare/html/) and visualized with TBtools [65].

### RNA-seq analysis

Transcriptome data including tissues (roots, stems, leaves, pistils, stamens, calyxes, petals and receptacles) under adverse stress (cold, heat, drought and salt) conditions (Genome sequencing project accession: PRJNA248163) and *V. dahliae* inoculation (Genome sequencing project accession: SRP118279) of *G. hirsutum* were downloaded from the NCBI Sequence Read Archive (SAR) database. The expression data were analyzed by standard analysis of the transcriptome on the raw data [66–68]. Log10 (TPM + 1) normalization was performed on the expression data. The standardized data were drawn with R-4.0.2 language.

### qRT-PCR

According to the cDNA information of the GhDUF668 genes, primers in the specific region were designed at the 5′ end or 3′ end of the gene sequence with Primer 5.0 software (Table S4). cDNA extracted from root tissue was used as the template, and the expression of candidate genes was detected with qRT-PCR. The GhUBQ7 gene was chosen as the reference gene for qRT-PCR analysis. Each entire experiment was repeated three times.

qRT-PCR was performed according to a previously reported method [30]. In brief, total RNA was isolated using a total RNA extraction kit (Tiangen, China). Then, 1 μg of total RNA after DNase I digestion was reverse transcribed into cDNA using 5× All-In-One Mastermix (Abm, Canada). Real-time PCR amplification was performed with an ABI 7500 Fast Real-Time PCR system. The cDNA was amplified using a BrightGreen 2× qPCR Mastermix (Abm, Canada) kit. The reaction program was a thermal cycling program at 94 °C for 30 s followed by 40 cycles of 95 °C for 5 s, 57 °C for 5 s, and 72 °C for 34 s. The relative RNA transcript levels of the candidate genes were calculated according to the 2^−ΔΔCT^ method [69].

### Protein interaction network analysis

A comparison of GhDUF668 gene was performed in the Arabidopsis information resource (https://www.arabidopsis.org/) by using homologous protein analysis. The homologous gene of GhDUF 668 in *Arabidopsis thaliana* was screened out and submitted to database of STRING (https://string-db.org/).And the relationship of protein-protein interaction network of *Arabidopsis thaliana* was further obtained from the database. Then, the homologous genes of *Gossypium hirsutum* were screened out by a comparison of the interaction genes from Arabidopsis in COTTONGEN (http://www.cottongen.org/) [55]. The software Cytoscape-3.8.2 was performed for visualization [70].

## Supplementary Information

**Additional file 1: Figure S1.** Distribution of the DUF668 domain in the DUF668 proteins of cotton.

**Additional file 2: Table S1.** Information on the DUF668 gene family in *G. barbadense, G. arboreum* and *G. raimondii.*

**Additional file 3: Table S2.** cotton DUF668 gene protein sequences.

**Additional file 4: Figure S2.** Conservative motifs of DUF668 gene family in *Arabidopsis thaliana, rice, G. hirsutum, G. barbadense, G. arboreum* and *G. raimondii.*

**Additional file 5: Figure S3.** Conservative motifs of DUF668 gene family in *G. hirsutum.*

**Additional file 6: Figure S4.** Interaction network of AT1G30755 proteins in Arabidopsi*s.*

**Additional file 7: Table S3.** cis-acting elements analysis of DUF668 family members in *G. hirsutum.*

**Additional file 8: Table S4.** All primers used in this study.

## Data Availability

The genome databases were downloaded from COTTONGEN (http://www.cottongen.org/) and RNA-seq (https://www.ncbi.nlm.nih.gov/sra/?term=). The datasets supporting the conclusions of this article are included in the article and its Additional files.
