# Supplementary material for: Genome-wide identification of the DUF668 gene family in cotton and expression profiling analysis of GhDUF668 in Gossypium hirsutum under adverse stress
Source: BMC Genomics. 2021 May 27;22:395. doi: 10.1186/s12864-021-07716-w (PMC8162019; doi:10.1186/s12864-021-07716-w)
Supplement: Supplementary file 1 — Additional file 1: Figure S1. Distribution of the DUF668 domain in the DUF668 proteins of cotton. [file 12864_2021_7716_MOESM1_ESM.pdf]

G. hirsutum

Gh_DUF668-01

Protein Classification

DUF668 and DUF3475 domain-containing protein (domain architecture ID 10571731)
DUF668 and DUF3475 domain-containing protein

Graphical summary Zoom to residue level show extra options »

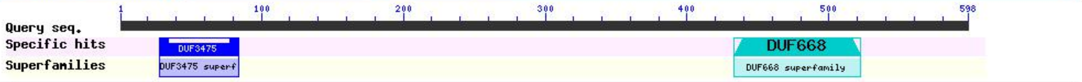

Search for similar domain architectures | Refine search

List of domain hits

Name	Accession	Description	Interval	E-value
[+] DUF668	pfam05003	Protein of unknown function (DUF668); Uncharacterized plant protein.	433-522	1.46e-48
[+] DUF3475	pfam11961	Domain of unknown function (DUF3475); This presumed domain is functionally uncharacterized. ...	28-84	1.24e-25

Gh_DUF668-02

Protein Classification

DUF668 and DUF3475 domain-containing protein (domain architecture ID 10571731)
DUF668 and DUF3475 domain-containing protein

Graphical summary Zoom to residue level show extra options »

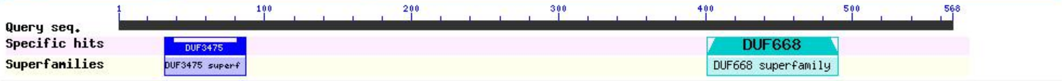

Search for similar domain architectures | Refine search

List of domain hits

Name	Accession	Description	Interval	E-value
[+] DUF668	pfam05003	Protein of unknown function (DUF668); Uncharacterized plant protein.	401-490	1.85e-44
[+] DUF3475	pfam11961	Domain of unknown function (DUF3475); This presumed domain is functionally uncharacterized. ...	32-87	8.74e-25

Gh_DUF668-03

Protein Classification

DUF668 and DUF3475 domain-containing protein (domain architecture ID 10571731)
DUF668 and DUF3475 domain-containing protein

Graphical summary Zoom to residue level show extra options »

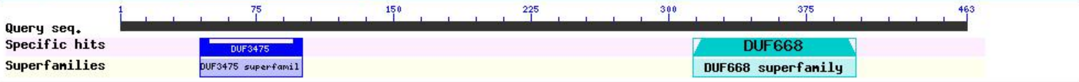

Search for similar domain architectures | Refine search

List of domain hits

Name	Accession	Description	Interval	E-value
[+] DUF668	pfam05003	Protein of unknown function (DUF668); Uncharacterized plant protein.	313-402	1.24e-29
[+] DUF3475	pfam11961	Domain of unknown function (DUF3475); This presumed domain is functionally uncharacterized. ...	44-100	1.23e-19

Gh_DUF668-04

Protein Classification

DUF668 and DUF3475 domain-containing protein (domain architecture ID 10571731)
DUF668 and DUF3475 domain-containing protein

Graphical summary Zoom to residue level show extra options »

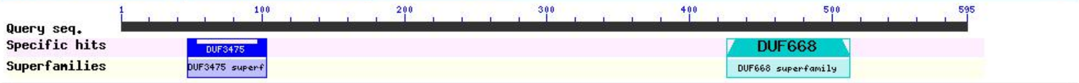

Search for similar domain architectures | Refine search

List of domain hits

Name	Accession	Description	Interval	E-value
[+] DUF668	pfam05003	Protein of unknown function (DUF668); Uncharacterized plant protein.	426-513	1.69e-49
[+] DUF3475	pfam11961	Domain of unknown function (DUF3475); This presumed domain is functionally uncharacterized. ...	47-103	1.02e-23

Gh_DUF668-05

Protein Classification

DUF668 and DUF3475 domain-containing protein (domain architecture ID 10571731)
DUF668 and DUF3475 domain-containing protein

Graphical summary

Zoom to residue level   show extra options »

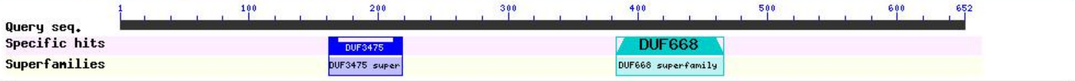

Search for similar domain architectures   Refine search

List of domain hits

Name	Accession	Description	Interval	E-value
[+] DUF668	pfam05003	Protein of unknown function (DUF668); Uncharacterized plant protein.	383-466	3.72e-46
[+] DUF3475	pfam11961	Domain of unknown function (DUF3475); This presumed domain is functionally uncharacterized. ...	162-218	1.90e-25

Gh_DUF668-06

Protein Classification

DUF668 domain-containing protein (domain architecture ID 10523325)
DUF668 domain-containing protein

Graphical summary

Zoom to residue level   show extra options »

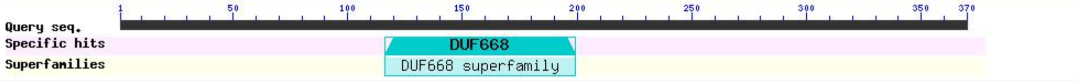

Search for similar domain architectures   Refine search

List of domain hits

Name	Accession	Description	Interval	E-value
[+] DUF668	pfam05003	Protein of unknown function (DUF668); Uncharacterized plant protein.	116-199	3.99e-41

Gh_DUF668-07

Protein Classification

DUF668 and DUF3475 domain-containing protein (domain architecture ID 10571731)
DUF668 and DUF3475 domain-containing protein

Graphical summary

Zoom to residue level   show extra options »

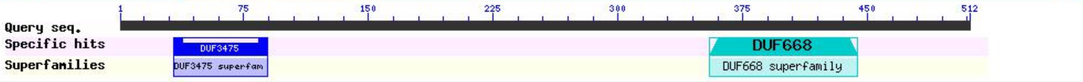

Search for similar domain architectures   Refine search

List of domain hits

Name	Accession	Description	Interval	E-value
[+] DUF668	pfam05003	Protein of unknown function (DUF668); Uncharacterized plant protein.	355-444	5.86e-29
[+] DUF3475	pfam11961	Domain of unknown function (DUF3475); This presumed domain is functionally uncharacterized. ...	33-89	5.81e-19

Gh_DUF668-08

Protein Classification

DUF668 and DUF3475 domain-containing protein (domain architecture ID 10571731)
DUF668 and DUF3475 domain-containing protein

Graphical summary

Zoom to residue level   show extra options »

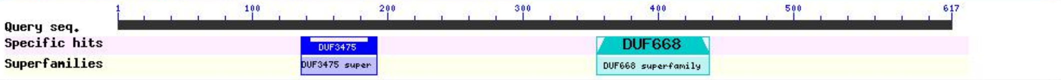

Search for similar domain architectures   Refine search

List of domain hits

Name	Accession	Description	Interval	E-value
[+] DUF668	pfam05003	Protein of unknown function (DUF668); Uncharacterized plant protein.	355-438	1.12e-39
[+] DUF3475	pfam11961	Domain of unknown function (DUF3475); This presumed domain is functionally uncharacterized. ...	136-192	4.99e-23

Gh_DUF668-09

Protein Classification

DUF668 and DUF3475 domain-containing protein (domain architecture ID 10571731)
DUF668 and DUF3475 domain-containing protein

Graphical summary Zoom to residue level show extra options »

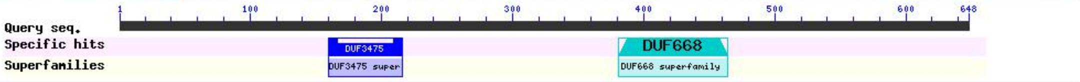

Search for similar domain architectures Refine search

List of domain hits

Name	Accession	Description	Interval	E-value
[+] DUF668	pfam05003	Protein of unknown function (DUF668); Uncharacterized plant protein.	381-464	3.78e-47
[+] DUF3475	pfam11961	Domain of unknown function (DUF3475); This presumed domain is functionally uncharacterized. ...	160-216	5.52e-26

Gh_DUF668-10

Protein Classification

DUF668 and DUF3475 domain-containing protein (domain architecture ID 10571731)
DUF668 and DUF3475 domain-containing protein

Graphical summary Zoom to residue level show extra options »

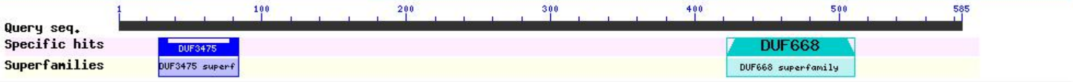

Search for similar domain architectures Refine search

List of domain hits

Name	Accession	Description	Interval	E-value
[+] DUF668	pfam05003	Protein of unknown function (DUF668); Uncharacterized plant protein.	422-511	5.82e-48
[+] DUF3475	pfam11961	Domain of unknown function (DUF3475); This presumed domain is functionally uncharacterized. ...	28-84	5.74e-23

Gh_DUF668-11

Protein Classification

DUF668 and DUF3475 domain-containing protein (domain architecture ID 10571731)
DUF668 and DUF3475 domain-containing protein

Graphical summary Zoom to residue level show extra options »

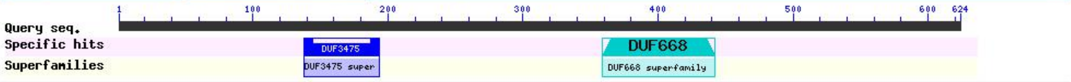

Search for similar domain architectures Refine search

List of domain hits

Name	Accession	Description	Interval	E-value
[+] DUF668	pfam05003	Protein of unknown function (DUF668); Uncharacterized plant protein.	359-442	9.28e-47
[+] DUF3475	pfam11961	Domain of unknown function (DUF3475); This presumed domain is functionally uncharacterized. ...	138-194	1.11e-21

Gh_DUF668-12

Protein Classification

DUF668 and DUF3475 domain-containing protein (domain architecture ID 10571731)
DUF668 and DUF3475 domain-containing protein

Graphical summary Zoom to residue level show extra options »

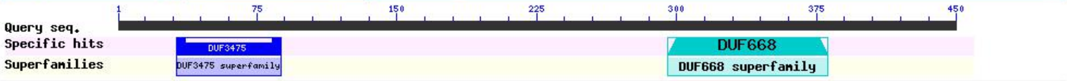

Search for similar domain architectures Refine search

List of domain hits

Name	Accession	Description	Interval	E-value
[+] DUF668	pfam05003	Protein of unknown function (DUF668); Uncharacterized plant protein.	295-381	2.16e-42
[+] DUF3475	pfam11961	Domain of unknown function (DUF3475); This presumed domain is functionally uncharacterized. ...	32-88	6.39e-22

Gh_DUF668-13

Protein Classification

DUF668 and DUF3475 domain-containing protein (domain architecture ID 10571731)
DUF668 and DUF3475 domain-containing protein

Graphical summary Zoom to residue level show extra options »

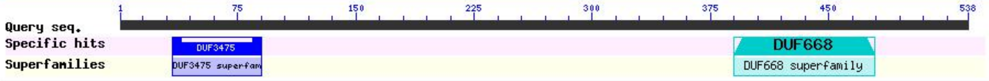

Search for similar domain architectures | Refine search

List of domain hits

Name	Accession	Description	Interval	E-value
[+] DUF668	pfam05003	Protein of unknown function (DUF668); Uncharacterized plant protein.	390-479	4.25e-41
[+] DUF3475	pfam11961	Domain of unknown function (DUF3475); This presumed domain is functionally uncharacterized. ...	34-90	7.99e-22

Gh_DUF668-14

Protein Classification

DUF668 and DUF3475 domain-containing protein (domain architecture ID 10571731)
DUF668 and DUF3475 domain-containing protein

Graphical summary Zoom to residue level show extra options »

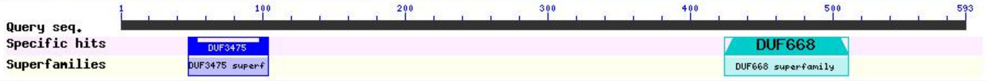

Search for similar domain architectures | Refine search

List of domain hits

Name	Accession	Description	Interval	E-value
[+] DUF668	pfam05003	Protein of unknown function (DUF668); Uncharacterized plant protein.	424-511	1.53e-49
[+] DUF3475	pfam11961	Domain of unknown function (DUF3475); This presumed domain is functionally uncharacterized. ...	48-104	6.88e-24

Gh_DUF668-15

Protein Classification

DUF668 and DUF3475 domain-containing protein (domain architecture ID 10571731)
DUF668 and DUF3475 domain-containing protein

Graphical summary Zoom to residue level show extra options »

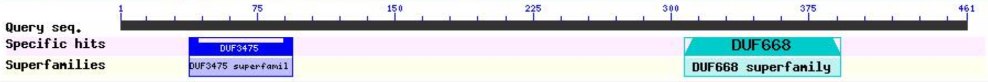

Search for similar domain architectures | Refine search

List of domain hits

Name	Accession	Description	Interval	E-value
[+] DUF668	pfam05003	Protein of unknown function (DUF668); Uncharacterized plant protein.	307-392	1.49e-44
[+] DUF3475	pfam11961	Domain of unknown function (DUF3475); This presumed domain is functionally uncharacterized. ...	38-94	4.53e-26

Gh_DUF668-16

Protein Classification

DUF668 and DUF3475 domain-containing protein (domain architecture ID 10571731)
DUF668 and DUF3475 domain-containing protein

Graphical summary Zoom to residue level show extra options »

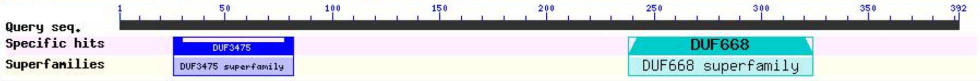

Search for similar domain architectures | Refine search

List of domain hits

Name	Accession	Description	Interval	E-value
[+] DUF668	pfam05003	Protein of unknown function (DUF668); Uncharacterized plant protein.	238-324	2.18e-41
[+] DUF3475	pfam11961	Domain of unknown function (DUF3475); This presumed domain is functionally uncharacterized. ...	26-82	3.26e-20

Gh_DUF668-17

Protein Classification

DUF668 and DUF3475 domain-containing protein (domain architecture ID 10571731)
DUF668 and DUF3475 domain-containing protein

Graphical summary

Zoom to residue level show extra options »

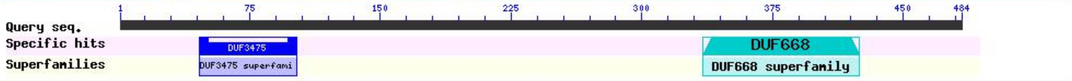

Search for similar domain architectures | Refine search

List of domain hits

Name	Accession	Description	Interval	E-value
[+] DUF668	pfam05003	Protein of unknown function (DUF668); Uncharacterized plant protein.	335-425	4.79e-27
[+] DUF3475	pfam11961	Domain of unknown function (DUF3475); This presumed domain is functionally uncharacterized. ...	46-102	1.91e-19

Gh_DUF668-18

Protein Classification

DUF668 and DUF3475 domain-containing protein (domain architecture ID 10571731)
DUF668 and DUF3475 domain-containing protein

Graphical summary

Zoom to residue level show extra options »

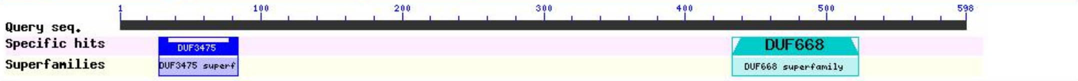

Search for similar domain architectures | Refine search

List of domain hits

Name	Accession	Description	Interval	E-value
[+] DUF668	pfam05003	Protein of unknown function (DUF668); Uncharacterized plant protein.	433-522	2.45e-48
[+] DUF3475	pfam11961	Domain of unknown function (DUF3475); This presumed domain is functionally uncharacterized. ...	28-84	1.38e-25

Gh_DUF668-19

Protein Classification

DUF668 and DUF3475 domain-containing protein (domain architecture ID 10571731)
DUF668 and DUF3475 domain-containing protein

Graphical summary

Zoom to residue level show extra options »

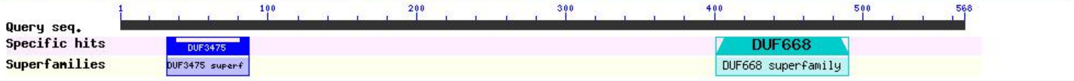

Search for similar domain architectures | Refine search

List of domain hits

Name	Accession	Description	Interval	E-value
[+] DUF668	pfam05003	Protein of unknown function (DUF668); Uncharacterized plant protein.	401-490	1.51e-44
[+] DUF3475	pfam11961	Domain of unknown function (DUF3475); This presumed domain is functionally uncharacterized. ...	32-87	3.01e-24

Gh_DUF668-20

Protein Classification

DUF668 and DUF3475 domain-containing protein (domain architecture ID 10571731)
DUF668 and DUF3475 domain-containing protein

Graphical summary

Zoom to residue level show extra options »

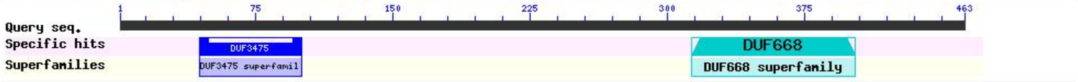

Search for similar domain architectures | Refine search

List of domain hits

Name	Accession	Description	Interval	E-value
[+] DUF668	pfam05003	Protein of unknown function (DUF668); Uncharacterized plant protein.	313-403	5.64e-28
[+] DUF3475	pfam11961	Domain of unknown function (DUF3475); This presumed domain is functionally uncharacterized. ...	44-100	1.74e-20

Gh_DUF668-21

Protein Classification

DUF668 and DUF3475 domain-containing protein (domain architecture ID 10571731)
DUF668 and DUF3475 domain-containing protein

Graphical summary Zoom to residue level show extra options »

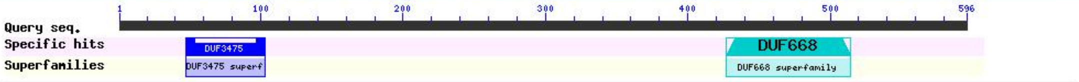

Search for similar domain architectures Refine search

List of domain hits

Name	Accession	Description	Interval	E-value
[+] DUF668	pfam05003	Protein of unknown function (DUF668); Uncharacterized plant protein.	427-514	2.42e-49
[+] DUF3475	pfam11961	Domain of unknown function (DUF3475); This presumed domain is functionally uncharacterized. ...	47-103	1.09e-23

Gh_DUF668-22

Protein Classification

DUF668 and DUF3475 domain-containing protein (domain architecture ID 10571731)
DUF668 and DUF3475 domain-containing protein

Graphical summary Zoom to residue level show extra options »

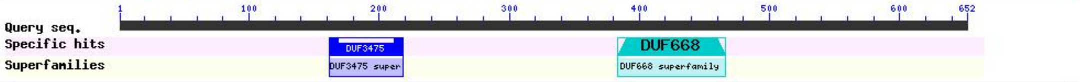

Search for similar domain architectures Refine search

List of domain hits

Name	Accession	Description	Interval	E-value
[+] DUF668	pfam05003	Protein of unknown function (DUF668); Uncharacterized plant protein.	383-466	3.72e-46
[+] DUF3475	pfam11961	Domain of unknown function (DUF3475); This presumed domain is functionally uncharacterized. ...	162-218	3.09e-24

Gh_DUF668-23

Protein Classification

DUF668 and DUF3475 domain-containing protein (domain architecture ID 10571731)
DUF668 and DUF3475 domain-containing protein

Graphical summary Zoom to residue level show extra options »

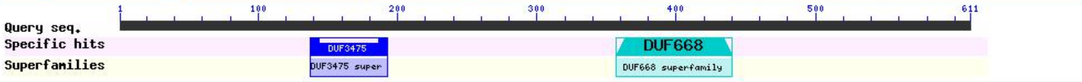

Search for similar domain architectures Refine search

List of domain hits

Name	Accession	Description	Interval	E-value
[+] DUF668	pfam05003	Protein of unknown function (DUF668); Uncharacterized plant protein.	357-440	2.38e-39
[+] DUF3475	pfam11961	Domain of unknown function (DUF3475); This presumed domain is functionally uncharacterized. ...	137-193	2.75e-22

Gh_DUF668-24

Protein Classification

DUF668 domain-containing protein (domain architecture ID 10523325)
DUF668 domain-containing protein

Graphical summary Zoom to residue level show extra options »

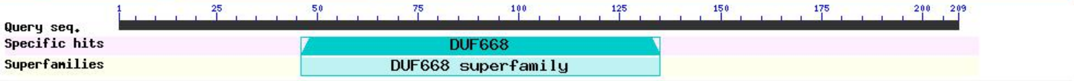

Search for similar domain architectures Refine search

List of domain hits

Name	Accession	Description	Interval	E-value
[+] DUF668	pfam05003	Protein of unknown function (DUF668); Uncharacterized plant protein.	46-135	4.05e-48

Gh_DUF668-25

Protein Classification

DUF668 and DUF3475 domain-containing protein (domain architecture ID 10571731)
DUF668 and DUF3475 domain-containing protein

Graphical summary

Zoom to residue level   show extra options »

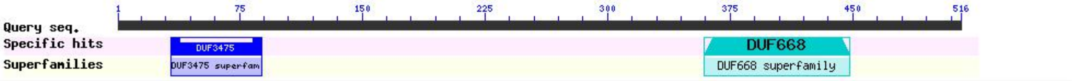

Search for similar domain architectures   Refine search

List of domain hits

Name	Accession	Description	Interval	E-value
[+] DUF668	pfam05003	Protein of unknown function (DUF668); Uncharacterized plant protein.	359-448	6.37e-31
[+] DUF3475	pfam11961	Domain of unknown function (DUF3475); This presumed domain is functionally uncharacterized. ...	33-89	7.80e-19

Gh_DUF668-26

Protein Classification

DUF668 and DUF3475 domain-containing protein (domain architecture ID 10571731)
DUF668 and DUF3475 domain-containing protein

Graphical summary

Zoom to residue level   show extra options »

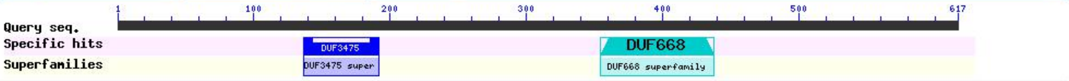

Search for similar domain architectures   Refine search

List of domain hits

Name	Accession	Description	Interval	E-value
[+] DUF668	pfam05003	Protein of unknown function (DUF668); Uncharacterized plant protein.	355-438	1.21e-39
[+] DUF3475	pfam11961	Domain of unknown function (DUF3475); This presumed domain is functionally uncharacterized. ...	137-192	3.87e-22

Gh_DUF668-27

Protein Classification

DUF668 and DUF3475 domain-containing protein (domain architecture ID 10571731)
DUF668 and DUF3475 domain-containing protein

Graphical summary

Zoom to residue level   show extra options »

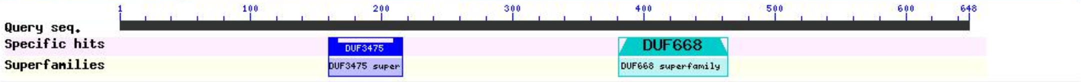

Search for similar domain architectures   Refine search

List of domain hits

Name	Accession	Description	Interval	E-value
[+] DUF668	pfam05003	Protein of unknown function (DUF668); Uncharacterized plant protein.	381-464	3.16e-45
[+] DUF3475	pfam11961	Domain of unknown function (DUF3475); This presumed domain is functionally uncharacterized. ...	160-216	4.54e-26

Gh_DUF668-28

Protein Classification

DUF668 and DUF3475 domain-containing protein (domain architecture ID 10571731)
DUF668 and DUF3475 domain-containing protein

Graphical summary

Zoom to residue level   show extra options »

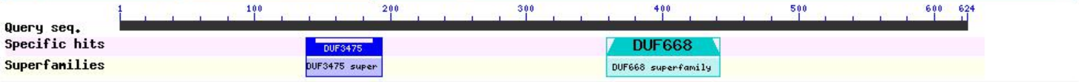

Search for similar domain architectures   Refine search

List of domain hits

Name	Accession	Description	Interval	E-value
[+] DUF668	pfam05003	Protein of unknown function (DUF668); Uncharacterized plant protein.	359-442	5.30e-45
[+] DUF3475	pfam11961	Domain of unknown function (DUF3475); This presumed domain is functionally uncharacterized. ...	138-194	1.63e-21

Gh_DUF668-29

Protein Classification

DUF668 and DUF3475 domain-containing protein (domain architecture ID 10571731)
DUF668 and DUF3475 domain-containing protein

Graphical summary

Zoom to residue level   show extra options »

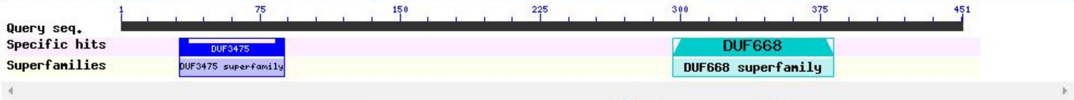

Search for similar domain architectures   Refine search

List of domain hits

Name	Accession	Description	Interval	E-value
[+] DUF668	pfam05003	Protein of unknown function (DUF668); Uncharacterized plant protein.	296-382	5.39e-44
[+] DUF3475	pfam11961	Domain of unknown function (DUF3475); This presumed domain is functionally uncharacterized. ...	32-88	2.32e-23

Gh_DUF668-30

Protein Classification

DUF668 and DUF3475 domain-containing protein (domain architecture ID 10571731)
DUF668 and DUF3475 domain-containing protein

Graphical summary

Zoom to residue level   show extra options »

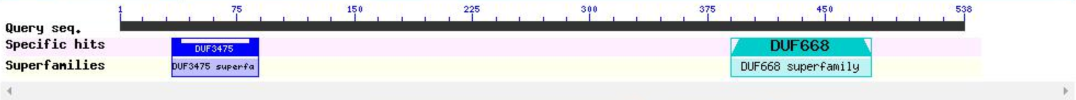

Search for similar domain architectures   Refine search

List of domain hits

Name	Accession	Description	Interval	E-value
[+] DUF668	pfam05003	Protein of unknown function (DUF668); Uncharacterized plant protein.	390-479	1.56e-37
[+] DUF3475	pfam11961	Domain of unknown function (DUF3475); This presumed domain is functionally uncharacterized. ...	34-89	1.22e-20

Gh_DUF668-31

Protein Classification

DUF668 and DUF3475 domain-containing protein (domain architecture ID 10571731)
DUF668 and DUF3475 domain-containing protein

Graphical summary

Zoom to residue level   show extra options »

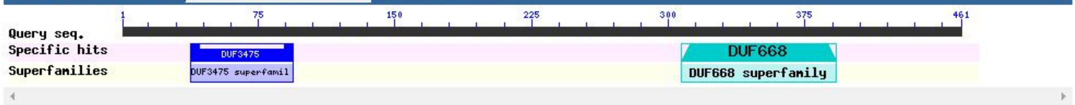

Search for similar domain architectures   Refine search

List of domain hits

Name	Accession	Description	Interval	E-value
[+] DUF668	pfam05003	Protein of unknown function (DUF668); Uncharacterized plant protein.	307-392	1.50e-44
[+] DUF3475	pfam11961	Domain of unknown function (DUF3475); This presumed domain is functionally uncharacterized. ...	38-94	2.74e-26

Gh_DUF668-32

Protein Classification

DUF3475 and DUF668 domain-containing protein (domain architecture ID 10571716)
DUF3475 and DUF668 domain-containing protein

Graphical summary

Zoom to residue level   show extra options »

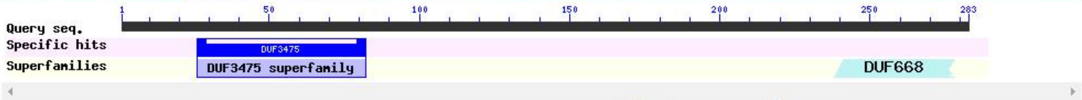

Search for similar domain architectures   Refine search

List of domain hits

Name	Accession	Description	Interval	E-value
[+] DUF3475	pfam11961	Domain of unknown function (DUF3475); This presumed domain is functionally uncharacterized. ...	26-82	3.65e-19
[+] DUF668 super family	cl04883	Protein of unknown function (DUF668); Uncharacterized plant protein.	238-278	6.87e-18

G. barbadense

Gb_DUF668-01

Protein Classification

DUF668 and DUF3475 domain-containing protein (domain architecture ID 10571731)
DUF668 and DUF3475 domain-containing protein

Graphical summary Zoom to residue level show extra options »

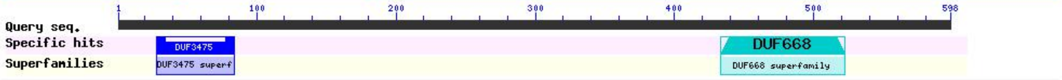

Search for similar domain architectures | Refine search

List of domain hits

Name	Accession	Description	Interval	E-value
[+] DUF668	pfam05003	Protein of unknown function (DUF668); Uncharacterized plant protein.	433-522	1.74e-48
[+] DUF3475	pfam11961	Domain of unknown function (DUF3475); This presumed domain is functionally uncharacterized. ...	28-84	1.60e-25

Gb_DUF668-02

Protein Classification

DUF668 and DUF3475 domain-containing protein (domain architecture ID 10571731)
DUF668 and DUF3475 domain-containing protein

Graphical summary Zoom to residue level show extra options »

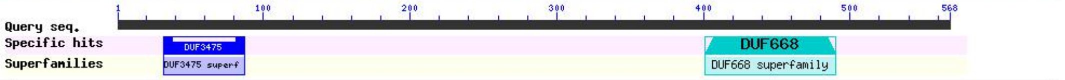

Search for similar domain architectures | Refine search

List of domain hits

Name	Accession	Description	Interval	E-value
[+] DUF668	pfam05003	Protein of unknown function (DUF668); Uncharacterized plant protein.	401-490	1.43e-44
[+] DUF3475	pfam11961	Domain of unknown function (DUF3475); This presumed domain is functionally uncharacterized. ...	32-87	8.57e-25

DUF668 and DUF3475 domain-containing protein (domain architecture ID 10571731)
DUF668 and DUF3475 domain-containing protein

Graphical summary Zoom to residue level show extra options »

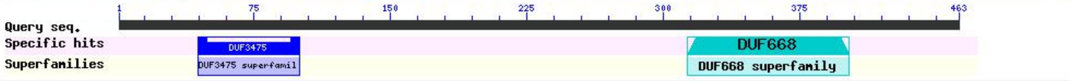

Search for similar domain architectures | Refine search

List of domain hits

Name	Accession	Description	Interval	E-value
[+] DUF668	pfam05003	Protein of unknown function (DUF668); Uncharacterized plant protein.	313-402	2.61e-27
[+] DUF3475	pfam11961	Domain of unknown function (DUF3475); This presumed domain is functionally uncharacterized. ...	44-100	3.03e-20

Gb_DUF668-04

Protein Classification

DUF668 and DUF3475 domain-containing protein (domain architecture ID 10571731)
DUF668 and DUF3475 domain-containing protein

Graphical summary Zoom to residue level show extra options »

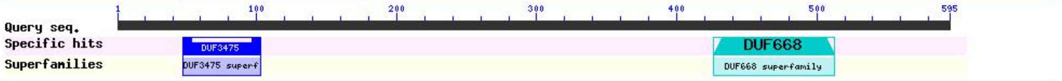

Search for similar domain architectures | Refine search

List of domain hits

Name	Accession	Description	Interval	E-value
[+] DUF668	pfam05003	Protein of unknown function (DUF668); Uncharacterized plant protein.	426-513	1.69e-49
[+] DUF3475	pfam11961	Domain of unknown function (DUF3475); This presumed domain is functionally uncharacterized. ...	47-103	1.02e-23

DUF668 and DUF3475 domain-containing protein (domain architecture ID 10571731)
DUF668 and DUF3475 domain-containing protein

Graphical summary Zoom to residue level show extra options »

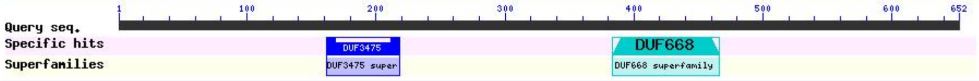

Search for similar domain architectures | Refine search

List of domain hits

Name	Accession	Description	Interval	E-value
[+] DUF668	pfam05003	Protein of unknown function (DUF668); Uncharacterized plant protein.	383-466	3.72e-46
[+] DUF3475	pfam11961	Domain of unknown function (DUF3475); This presumed domain is functionally uncharacterized. ...	162-218	2.10e-25

Gb_DUF668-06

Protein Classification

DUF668 and DUF3475 domain-containing protein (domain architecture ID 10571731)
DUF668 and DUF3475 domain-containing protein

Graphical summary Zoom to residue level show extra options »

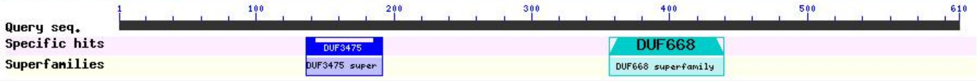

Search for similar domain architectures | Refine search

List of domain hits

Name	Accession	Description	Interval	E-value
[+] DUF668	pfam05003	Protein of unknown function (DUF668); Uncharacterized plant protein.	356-439	2.07e-40
[+] DUF3475	pfam11961	Domain of unknown function (DUF3475); This presumed domain is functionally uncharacterized. ...	136-192	1.65e-20

Gb_DUF668-07

Protein Classification

DUF668 and DUF3475 domain-containing protein (domain architecture ID 10571731)
DUF668 and DUF3475 domain-containing protein

Graphical summary Zoom to residue level show extra options »

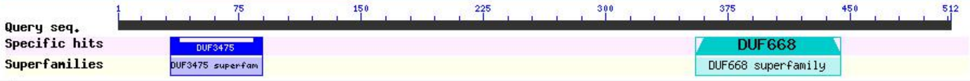

Search for similar domain architectures | Refine search

List of domain hits

Name	Accession	Description	Interval	E-value
[+] DUF668	pfam05003	Protein of unknown function (DUF668); Uncharacterized plant protein.	355-444	5.86e-29
[+] DUF3475	pfam11961	Domain of unknown function (DUF3475); This presumed domain is functionally uncharacterized. ...	33-89	6.41e-19

Gb_DUF668-08

Protein Classification

DUF668 and DUF3475 domain-containing protein (domain architecture ID 10571731)
DUF668 and DUF3475 domain-containing protein

Graphical summary Zoom to residue level show extra options »

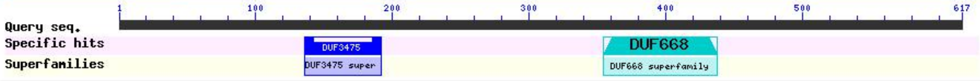

Search for similar domain architectures | Refine search

List of domain hits

Name	Accession	Description	Interval	E-value
[+] DUF668	pfam05003	Protein of unknown function (DUF668); Uncharacterized plant protein.	355-438	1.12e-39
[+] DUF3475	pfam11961	Domain of unknown function (DUF3475); This presumed domain is functionally uncharacterized. ...	136-192	4.03e-23

Gb_DUF668-09

Protein Classification

DUF668 and DUF3475 domain-containing protein (domain architecture ID 10571731)
DUF668 and DUF3475 domain-containing protein

Graphical summary Zoom to residue level show extra options »

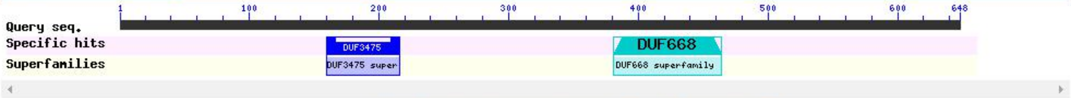

Search for similar domain architectures | Refine search

List of domain hits

Name	Accession	Description	Interval	E-value
[+] DUF668	pfam05003	Protein of unknown function (DUF668); Uncharacterized plant protein.	381-464	3.78e-47
[+] DUF3475	pfam11961	Domain of unknown function (DUF3475); This presumed domain is functionally uncharacterized. ...	160-216	2.21e-25

Gb_DUF668-10

Protein Classification

DUF668 and DUF3475 domain-containing protein (domain architecture ID 10571731)
DUF668 and DUF3475 domain-containing protein

Graphical summary Zoom to residue level show extra options »

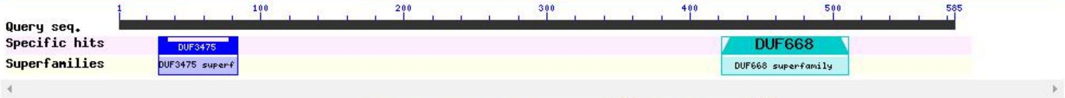

Search for similar domain architectures | Refine search

List of domain hits

Name	Accession	Description	Interval	E-value
[+] DUF668	pfam05003	Protein of unknown function (DUF668); Uncharacterized plant protein.	422-511	6.46e-48
[+] DUF3475	pfam11961	Domain of unknown function (DUF3475); This presumed domain is functionally uncharacterized. ...	28-84	6.71e-23

Gb_DUF668-11

Protein Classification

DUF668 and DUF3475 domain-containing protein (domain architecture ID 10571731)
DUF668 and DUF3475 domain-containing protein

Graphical summary Zoom to residue level show extra options »

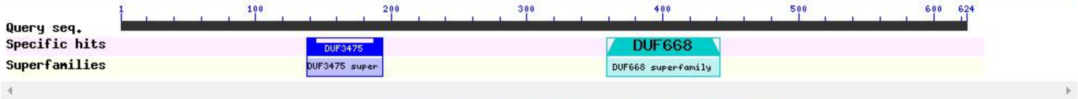

Search for similar domain architectures | Refine search

List of domain hits

Name	Accession	Description	Interval	E-value
[+] DUF668	pfam05003	Protein of unknown function (DUF668); Uncharacterized plant protein.	359-442	9.28e-47
[+] DUF3475	pfam11961	Domain of unknown function (DUF3475); This presumed domain is functionally uncharacterized. ...	138-194	9.48e-21

Gb_DUF668-12

Protein Classification

DUF668 and DUF3475 domain-containing protein (domain architecture ID 10571731)
DUF668 and DUF3475 domain-containing protein

Graphical summary Zoom to residue level show extra options »

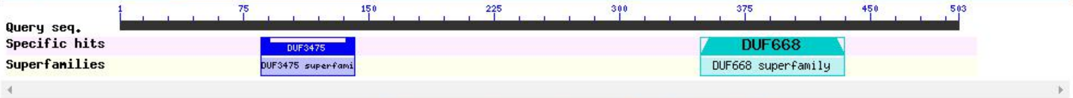

Search for similar domain architectures | Refine search

List of domain hits

Name	Accession	Description	Interval	E-value
[+] DUF668	pfam05003	Protein of unknown function (DUF668); Uncharacterized plant protein.	348-434	5.04e-42
[+] DUF3475	pfam11961	Domain of unknown function (DUF3475); This presumed domain is functionally uncharacterized. ...	85-141	8.64e-23

Gb_DUF668-13

Protein Classification

DUF668 and DUF3475 domain-containing protein (domain architecture ID 10571731)
DUF668 and DUF3475 domain-containing protein

Graphical summary

Zoom to residue level show extra options »

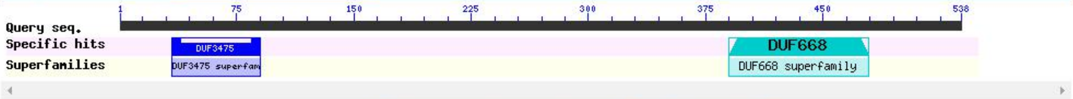

Search for similar domain architectures Refine search

List of domain hits

Name	Accession	Description	Interval	E-value
[+] DUF668	pfam05003	Protein of unknown function (DUF668); Uncharacterized plant protein.	390-479	1.18e-40
[+] DUF3475	pfam11961	Domain of unknown function (DUF3475); This presumed domain is functionally uncharacterized. ...	34-90	9.81e-22

Gb_DUF668-14

Protein Classification

DUF668 and DUF3475 domain-containing protein (domain architecture ID 10571731)
DUF668 and DUF3475 domain-containing protein

Graphical summary

Zoom to residue level show extra options »

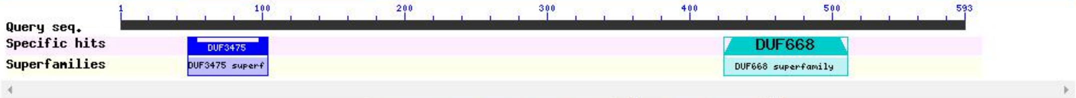

Search for similar domain architectures Refine search

List of domain hits

Name	Accession	Description	Interval	E-value
[+] DUF668	pfam05003	Protein of unknown function (DUF668); Uncharacterized plant protein.	424-511	4.30e-48
[+] DUF3475	pfam11961	Domain of unknown function (DUF3475); This presumed domain is functionally uncharacterized. ...	48-104	8.04e-24

Gb_DUF668-15

Protein Classification

DUF668 and DUF3475 domain-containing protein (domain architecture ID 10571731)
DUF668 and DUF3475 domain-containing protein

Graphical summary

Zoom to residue level show extra options »

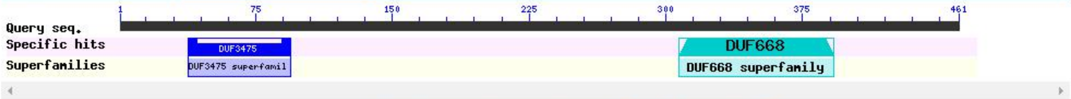

Search for similar domain architectures Refine search

List of domain hits

Name	Accession	Description	Interval	E-value
[+] DUF668	pfam05003	Protein of unknown function (DUF668); Uncharacterized plant protein.	307-392	1.52e-44
[+] DUF3475	pfam11961	Domain of unknown function (DUF3475); This presumed domain is functionally uncharacterized. ...	38-94	5.17e-26

Gb_DUF668-16

Protein Classification

DUF668 and DUF3475 domain-containing protein (domain architecture ID 10571731)
DUF668 and DUF3475 domain-containing protein

Graphical summary

Zoom to residue level show extra options »

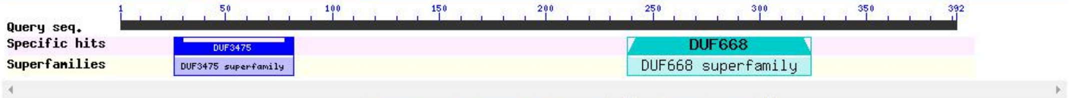

Search for similar domain architectures Refine search

List of domain hits

Name	Accession	Description	Interval	E-value
[+] DUF668	pfam05003	Protein of unknown function (DUF668); Uncharacterized plant protein.	238-324	2.15e-41
[+] DUF3475	pfam11961	Domain of unknown function (DUF3475); This presumed domain is functionally uncharacterized. ...	26-82	3.13e-20

Gb_DUF668-17

Protein Classification

DUF668 and DUF3475 domain-containing protein (domain architecture ID 10571731)
DUF668 and DUF3475 domain-containing protein

Graphical summary

Zoom to residue level | show extra options »

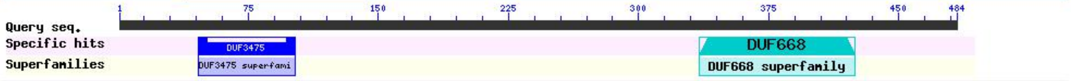

Search for similar domain architectures | Refine search

List of domain hits

Name	Accession	Description	Interval	E-value
[+] DUF668	pfam05003	Protein of unknown function (DUF668); Uncharacterized plant protein.	335-425	1.88e-28
[+] DUF3475	pfam11961	Domain of unknown function (DUF3475); This presumed domain is functionally uncharacterized. ...	46-102	1.97e-19

Gb_DUF668-18

Protein Classification

DUF668 and DUF3475 domain-containing protein (domain architecture ID 10571731)
DUF668 and DUF3475 domain-containing protein

Graphical summary

Zoom to residue level | show extra options »

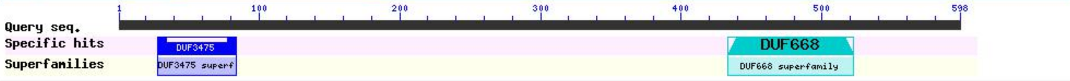

Search for similar domain architectures | Refine search

List of domain hits

Name	Accession	Description	Interval	E-value
[+] DUF668	pfam05003	Protein of unknown function (DUF668); Uncharacterized plant protein.	433-522	2.50e-48
[+] DUF3475	pfam11961	Domain of unknown function (DUF3475); This presumed domain is functionally uncharacterized. ...	28-84	1.39e-25

Gb_DUF668-19

Protein Classification

DUF668 and DUF3475 domain-containing protein (domain architecture ID 10571731)
DUF668 and DUF3475 domain-containing protein

Graphical summary

Zoom to residue level | show extra options »

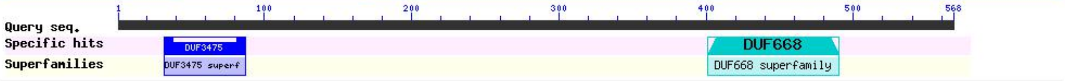

Search for similar domain architectures | Refine search

List of domain hits

Name	Accession	Description	Interval	E-value
[+] DUF668	pfam05003	Protein of unknown function (DUF668); Uncharacterized plant protein.	401-490	1.43e-44
[+] DUF3475	pfam11961	Domain of unknown function (DUF3475); This presumed domain is functionally uncharacterized. ...	32-87	2.33e-24

Gb_DUF668-20

Protein Classification

DUF668 and DUF3475 domain-containing protein (domain architecture ID 10571731)
DUF668 and DUF3475 domain-containing protein

Graphical summary

Zoom to residue level | show extra options »

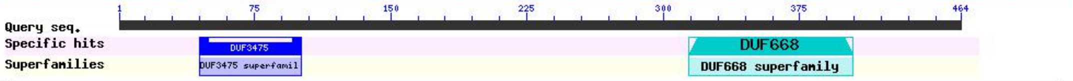

Search for similar domain architectures | Refine search

List of domain hits

Name	Accession	Description	Interval	E-value
[+] DUF668	pfam05003	Protein of unknown function (DUF668); Uncharacterized plant protein.	314-404	3.37e-30
[+] DUF3475	pfam11961	Domain of unknown function (DUF3475); This presumed domain is functionally uncharacterized. ...	45-101	1.11e-20

Gb_DUF668-21

Protein Classification

DUF668 and DUF3475 domain-containing protein (domain architecture ID 10571731)
DUF668 and DUF3475 domain-containing protein

Graphical summary Zoom to residue level show extra options »

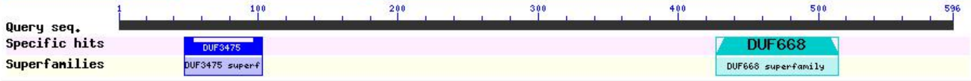

Search for similar domain architectures | Refine search

List of domain hits

Name	Accession	Description	Interval	E-value
[+] DUF668	pfam05003	Protein of unknown function (DUF668); Uncharacterized plant protein.	427-514	3.85e-49
[+] DUF3475	pfam11961	Domain of unknown function (DUF3475); This presumed domain is functionally uncharacterized. ...	47-103	1.00e-23

Gb_DUF668-22

Protein Classification

DUF668 and DUF3475 domain-containing protein (domain architecture ID 10571731)
DUF668 and DUF3475 domain-containing protein

Graphical summary Zoom to residue level show extra options »

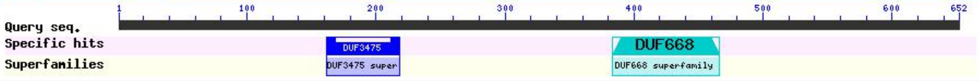

Search for similar domain architectures | Refine search

List of domain hits

Name	Accession	Description	Interval	E-value
[+] DUF668	pfam05003	Protein of unknown function (DUF668); Uncharacterized plant protein.	383-466	3.72e-46
[+] DUF3475	pfam11961	Domain of unknown function (DUF3475); This presumed domain is functionally uncharacterized. ...	162-218	2.94e-24

Gb_DUF668-23

Protein Classification

DUF668 and DUF3475 domain-containing protein (domain architecture ID 10571731)
DUF668 and DUF3475 domain-containing protein

Graphical summary Zoom to residue level show extra options »

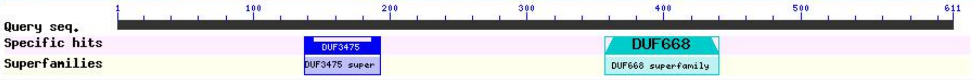

Search for similar domain architectures | Refine search

List of domain hits

Name	Accession	Description	Interval	E-value
[+] DUF668	pfam05003	Protein of unknown function (DUF668); Uncharacterized plant protein.	357-440	2.30e-39
[+] DUF3475	pfam11961	Domain of unknown function (DUF3475); This presumed domain is functionally uncharacterized. ...	137-193	2.64e-22

Gb_DUF668-24

Protein Classification

DUF668 domain-containing protein (domain architecture ID 10523325)
DUF668 domain-containing protein

Graphical summary Zoom to residue level show extra options »

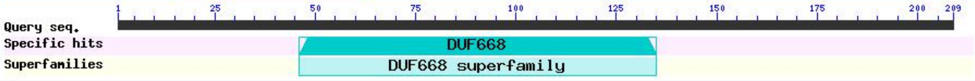

Search for similar domain architectures | Refine search

List of domain hits

Name	Accession	Description	Interval	E-value
[+] DUF668	pfam05003	Protein of unknown function (DUF668); Uncharacterized plant protein.	46-135	4.05e-48

Gb_DUF668-25

Protein Classification

DUF668 and DUF3475 domain-containing protein (domain architecture ID 10571731)
DUF668 and DUF3475 domain-containing protein

Graphical summary Zoom to residue level show extra options »

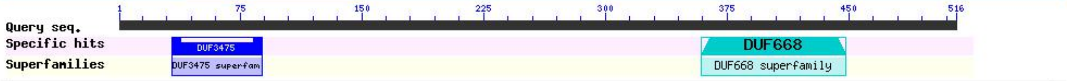

Search for similar domain architectures | Refine search

List of domain hits

Name	Accession	Description	Interval	E-value
[+] DUF668	pfam05003	Protein of unknown function (DUF668); Uncharacterized plant protein.	359-448	6.37e-31
[+] DUF3475	pfam11961	Domain of unknown function (DUF3475); This presumed domain is functionally uncharacterized. ...	33-89	7.80e-19

Gb_DUF668-26

Protein Classification

DUF668 and DUF3475 domain-containing protein (domain architecture ID 10571731)
DUF668 and DUF3475 domain-containing protein

Graphical summary Zoom to residue level show extra options »

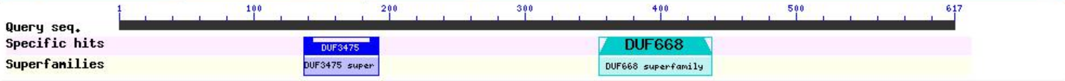

Search for similar domain architectures | Refine search

List of domain hits

Name	Accession	Description	Interval	E-value
[+] DUF668	pfam05003	Protein of unknown function (DUF668); Uncharacterized plant protein.	355-438	1.21e-39
[+] DUF3475	pfam11961	Domain of unknown function (DUF3475); This presumed domain is functionally uncharacterized. ...	137-192	4.31e-22

Gb_DUF668-27

Protein Classification

DUF668 and DUF3475 domain-containing protein (domain architecture ID 10571731)
DUF668 and DUF3475 domain-containing protein

Graphical summary Zoom to residue level show extra options »

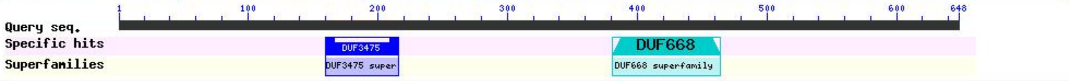

Search for similar domain architectures | Refine search

List of domain hits

Name	Accession	Description	Interval	E-value
[+] DUF668	pfam05003	Protein of unknown function (DUF668); Uncharacterized plant protein.	381-464	3.16e-45
[+] DUF3475	pfam11961	Domain of unknown function (DUF3475); This presumed domain is functionally uncharacterized. ...	160-216	5.47e-26

Gb_DUF668-28

Protein Classification

DUF668 and DUF3475 domain-containing protein (domain architecture ID 10571731)
DUF668 and DUF3475 domain-containing protein

Graphical summary Zoom to residue level show extra options »

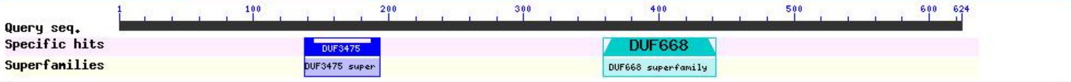

Search for similar domain architectures | Refine search

List of domain hits

Name	Accession	Description	Interval	E-value
[+] DUF668	pfam05003	Protein of unknown function (DUF668); Uncharacterized plant protein.	359-442	4.94e-45
[+] DUF3475	pfam11961	Domain of unknown function (DUF3475); This presumed domain is functionally uncharacterized. ...	138-194	1.13e-22

Gb_DUF668-29

Protein Classification

DUF668 and DUF3475 domain-containing protein (domain architecture ID 10571731)
DUF668 and DUF3475 domain-containing protein

Graphical summary Zoom to residue level show extra options »

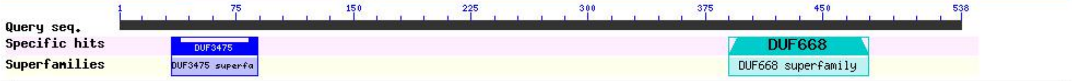

Search for similar domain architectures | Refine search

List of domain hits

Name	Accession	Description	Interval	E-value
[+] DUF668	pfam05003	Protein of unknown function (DUF668); Uncharacterized plant protein.	390-479	1.60e-37
[+] DUF3475	pfam11961	Domain of unknown function (DUF3475); This presumed domain is functionally uncharacterized. ...	34-89	1.10e-20

Gb_DUF668-30

Protein Classification

DUF668 and DUF3475 domain-containing protein (domain architecture ID 10571731)
DUF668 and DUF3475 domain-containing protein

Graphical summary Zoom to residue level show extra options »

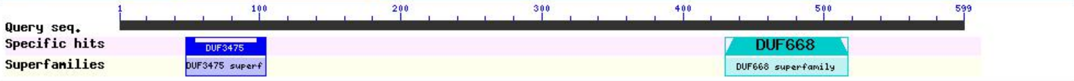

Search for similar domain architectures | Refine search

List of domain hits

Name	Accession	Description	Interval	E-value
[+] DUF668	pfam05003	Protein of unknown function (DUF668); Uncharacterized plant protein.	430-517	9.78e-49
[+] DUF3475	pfam11961	Domain of unknown function (DUF3475); This presumed domain is functionally uncharacterized. ...	48-104	1.65e-23

Gb_DUF668-31

Protein Classification

DUF668 and DUF3475 domain-containing protein (domain architecture ID 10571731)
DUF668 and DUF3475 domain-containing protein

Graphical summary Zoom to residue level show extra options »

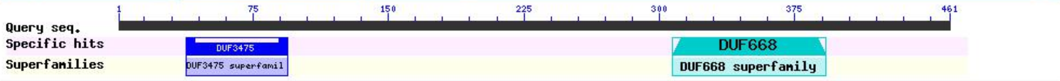

Search for similar domain architectures | Refine search

List of domain hits

Name	Accession	Description	Interval	E-value
[+] DUF668	pfam05003	Protein of unknown function (DUF668); Uncharacterized plant protein.	307-392	1.50e-44
[+] DUF3475	pfam11961	Domain of unknown function (DUF3475); This presumed domain is functionally uncharacterized. ...	38-94	2.74e-26

Gb_DUF668-32

Protein Classification

DUF668 and DUF3475 domain-containing protein (domain architecture ID 10571731)
DUF668 and DUF3475 domain-containing protein

Graphical summary Zoom to residue level show extra options »

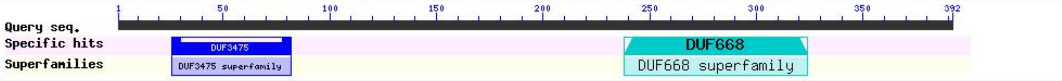

Search for similar domain architectures | Refine search

List of domain hits

Name	Accession	Description	Interval	E-value
[+] DUF668	pfam05003	Protein of unknown function (DUF668); Uncharacterized plant protein.	238-324	1.09e-41
[+] DUF3475	pfam11961	Domain of unknown function (DUF3475); This presumed domain is functionally uncharacterized. ...	26-82	1.83e-18

Gb_DUF668-33

Protein Classification

DUF668 domain-containing protein (domain architecture ID 10523325)
DUF668 domain-containing protein

Graphical summary Zoom to residue level show extra options »

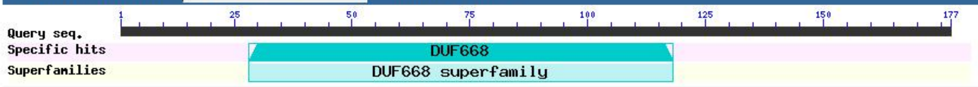

Search for similar domain architectures | Refine search

List of domain hits

Name	Accession	Description	Interval	E-value
[+] DUF668	pfam05003	Protein of unknown function (DUF668); Uncharacterized plant protein.	28-118	7.59e-33

References:

G. arboreum

Ga_DUF668-01

Protein Classification

DUF668 and DUF3475 domain-containing protein (domain architecture ID 10571731)
DUF668 and DUF3475 domain-containing protein

Graphical summary Zoom to residue level show extra options »

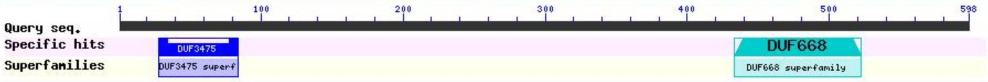

Search for similar domain architectures | Refine search

List of domain hits

Name	Accession	Description	Interval	E-value
[+] DUF668	pfam05003	Protein of unknown function (DUF668); Uncharacterized plant protein.	433-522	2.06e-47
[+] DUF3475	pfam11961	Domain of unknown function (DUF3475); This presumed domain is functionally uncharacterized. ...	28-84	1.38e-25

Ga_DUF668-02

Protein Classification

DUF668 and DUF3475 domain-containing protein (domain architecture ID 10571731)
DUF668 and DUF3475 domain-containing protein

Graphical summary Zoom to residue level show extra options »

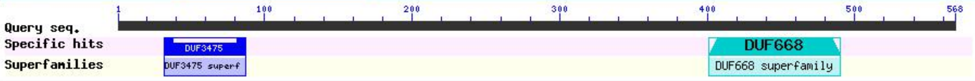

Search for similar domain architectures | Refine search

List of domain hits

Name	Accession	Description	Interval	E-value
[+] DUF668	pfam05003	Protein of unknown function (DUF668); Uncharacterized plant protein.	401-490	1.62e-44
[+] DUF3475	pfam11961	Domain of unknown function (DUF3475); This presumed domain is functionally uncharacterized. ...	32-87	2.31e-24

Ga_DUF668-03

Protein Classification

DUF668 and DUF3475 domain-containing protein (domain architecture ID 10571731)
DUF668 and DUF3475 domain-containing protein

Graphical summary Zoom to residue level show extra options »

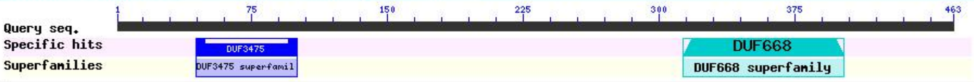

Search for similar domain architectures | Refine search

List of domain hits

Name	Accession	Description	Interval	E-value
[+] DUF668	pfam05003	Protein of unknown function (DUF668); Uncharacterized plant protein.	313-402	6.41e-29
[+] DUF3475	pfam11961	Domain of unknown function (DUF3475); This presumed domain is functionally uncharacterized. ...	44-100	1.36e-20

Ga_DUF668-04

Protein Classification

DUF668 and DUF3475 domain-containing protein (domain architecture ID 10571731)
DUF668 and DUF3475 domain-containing protein

Graphical summary Zoom to residue level show extra options »

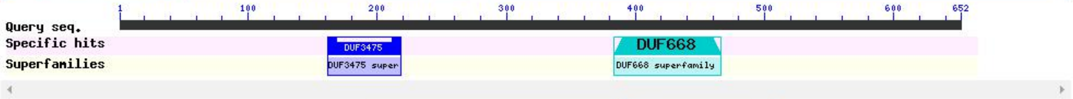

Search for similar domain architectures | Refine search

List of domain hits

Name	Accession	Description	Interval	E-value
[+] DUF668	pfam05003	Protein of unknown function (DUF668); Uncharacterized plant protein.	383-466	3.72e-46
[+] DUF3475	pfam11961	Domain of unknown function (DUF3475); This presumed domain is functionally uncharacterized. ...	162-218	2.12e-25

Ga_DUF668-05

Protein Classification

DUF668 and DUF3475 domain-containing protein (domain architecture ID 10571731)
DUF668 and DUF3475 domain-containing protein

Graphical summary Zoom to residue level show extra options »

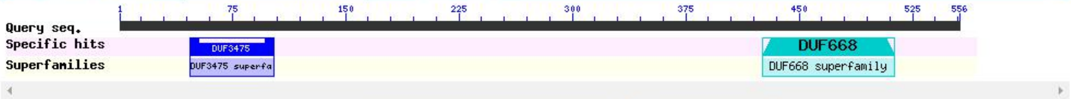

Search for similar domain architectures | Refine search

List of domain hits

Name	Accession	Description	Interval	E-value
[+] DUF668	pfam05003	Protein of unknown function (DUF668); Uncharacterized plant protein.	426-513	9.35e-50
[+] DUF3475	pfam11961	Domain of unknown function (DUF3475); This presumed domain is functionally uncharacterized. ...	47-103	1.40e-23

Ga_DUF668-06

Graphical summary Zoom to residue level show extra options »

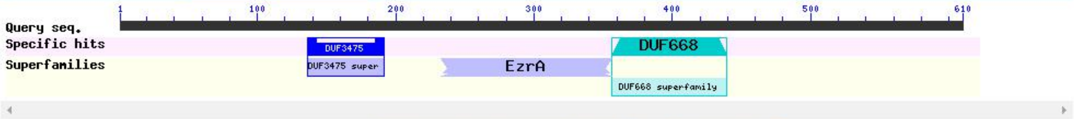

Search for similar domain architectures | Refine search

List of domain hits

Name	Accession	Description	Interval	E-value
[+] DUF668	pfam05003	Protein of unknown function (DUF668); Uncharacterized plant protein.	356-439	1.80e-40
[+] DUF3475	pfam11961	Domain of unknown function (DUF3475); This presumed domain is functionally uncharacterized. ...	136-192	8.50e-22
[+] EzrA super family	cl38199	Septation ring formation regulator, EzrA; During the bacterial cell cycle, the tubulin-like ...	233-356	8.99e-04

Ga_DUF668-07

Protein Classification

DUF668 and DUF3475 domain-containing protein (domain architecture ID 10571731)
DUF668 and DUF3475 domain-containing protein

Graphical summary Zoom to residue level show extra options »

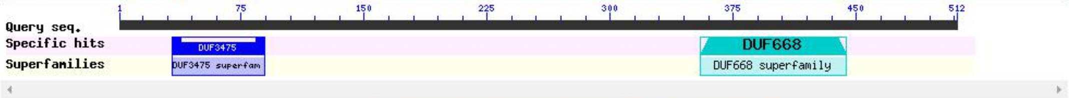

Search for similar domain architectures | Refine search

List of domain hits

Name	Accession	Description	Interval	E-value
[+] DUF668	pfam05003	Protein of unknown function (DUF668); Uncharacterized plant protein.	355-444	5.86e-29
[+] DUF3475	pfam11961	Domain of unknown function (DUF3475); This presumed domain is functionally uncharacterized. ...	33-89	5.54e-19

Ga_DUF668-08

Protein Classification

DUF668 and DUF3475 domain-containing protein (domain architecture ID 10571731)
DUF668 and DUF3475 domain-containing protein

Graphical summary Zoom to residue level show extra options »

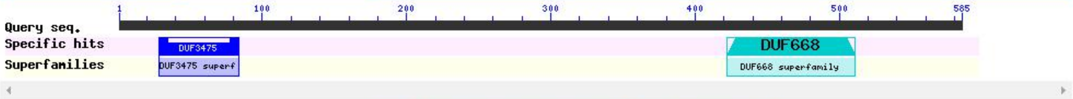

Search for similar domain architectures | Refine search

List of domain hits

Name	Accession	Description	Interval	E-value
[+] DUF668	pfam05003	Protein of unknown function (DUF668); Uncharacterized plant protein.	422-511	5.41e-48
[+] DUF3475	pfam11961	Domain of unknown function (DUF3475); This presumed domain is functionally uncharacterized. ...	28-84	6.03e-23

Ga_DUF668-09

Protein Classification

DUF668 and DUF3475 domain-containing protein (domain architecture ID 10571731)
DUF668 and DUF3475 domain-containing protein

Graphical summary Zoom to residue level show extra options »

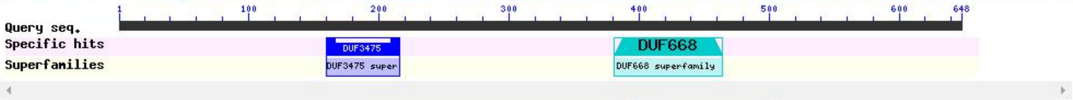

Search for similar domain architectures Refine search

List of domain hits

Name	Accession	Description	Interval	E-value
[+] DUF668	pfam05003	Protein of unknown function (DUF668); Uncharacterized plant protein.	381-464	3.78e-47
[+] DUF3475	pfam11961	Domain of unknown function (DUF3475); This presumed domain is functionally uncharacterized. ...	160-216	5.80e-26

Ga_DUF668-10

Graphical summary Zoom to residue level show extra options »

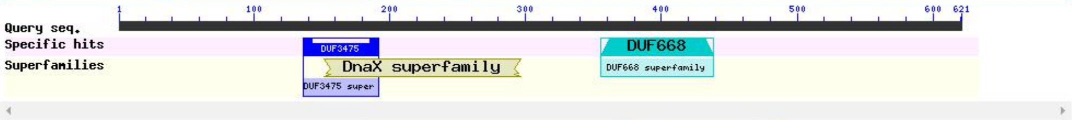

Search for similar domain architectures Refine search

List of domain hits

Name	Accession	Description	Interval	E-value
[+] DUF668	pfam05003	Protein of unknown function (DUF668); Uncharacterized plant protein.	355-438	1.16e-39
[+] DUF3475	pfam11961	Domain of unknown function (DUF3475); This presumed domain is functionally uncharacterized. ...	136-192	5.55e-23
[+] DnaX super family	cl39080	DNA polymerase III, gamma/tau subunits [Replication, recombination and repair];	152-297	6.30e-03

Ga_DUF668-11

Protein Classification

DUF668 and DUF3475 domain-containing protein (domain architecture ID 10571731)
DUF668 and DUF3475 domain-containing protein

Graphical summary Zoom to residue level show extra options »

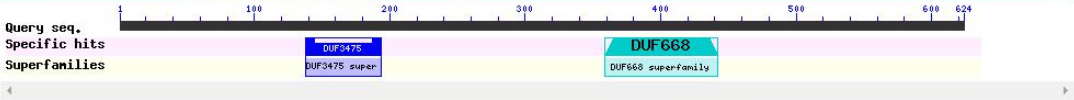

Search for similar domain architectures Refine search

List of domain hits

Name	Accession	Description	Interval	E-value
[+] DUF668	pfam05003	Protein of unknown function (DUF668); Uncharacterized plant protein.	359-442	9.28e-47
[+] DUF3475	pfam11961	Domain of unknown function (DUF3475); This presumed domain is functionally uncharacterized. ...	138-194	9.42e-22

Ga_DUF668-12

Protein Classification

DUF668 and DUF3475 domain-containing protein (domain architecture ID 10571731)
DUF668 and DUF3475 domain-containing protein

Graphical summary Zoom to residue level show extra options »

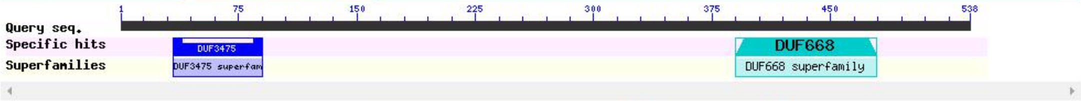

Search for similar domain architectures Refine search

List of domain hits

Name	Accession	Description	Interval	E-value
[+] DUF668	pfam05003	Protein of unknown function (DUF668); Uncharacterized plant protein.	390-479	1.38e-40
[+] DUF3475	pfam11961	Domain of unknown function (DUF3475); This presumed domain is functionally uncharacterized. ...	34-90	1.30e-21

Ga_DUF668-13

Protein Classification

DUF668 and DUF3475 domain-containing protein (domain architecture ID 10571731)
DUF668 and DUF3475 domain-containing protein

Graphical summary Zoom to residue level show extra options »

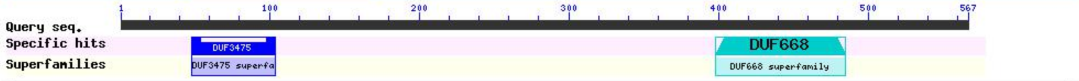

Search for similar domain architectures | Refine search

List of domain hits

Name	Accession	Description	Interval	E-value
[+] DUF668	pfam05003	Protein of unknown function (DUF668); Uncharacterized plant protein.	398-485	1.92e-49
[+] DUF3475	pfam11961	Domain of unknown function (DUF3475); This presumed domain is functionally uncharacterized. ...	48-104	7.46e-24

Ga_DUF668-14

Protein Classification

DUF668 and DUF3475 domain-containing protein (domain architecture ID 10571731)
DUF668 and DUF3475 domain-containing protein

Graphical summary Zoom to residue level show extra options »

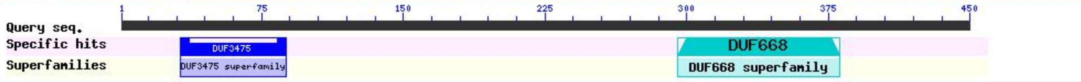

Search for similar domain architectures | Refine search

List of domain hits

Name	Accession	Description	Interval	E-value
[+] DUF668	pfam05003	Protein of unknown function (DUF668); Uncharacterized plant protein.	295-381	4.10e-42
[+] DUF3475	pfam11961	Domain of unknown function (DUF3475); This presumed domain is functionally uncharacterized. ...	32-88	7.68e-23

Ga_DUF668-15

Protein Classification

DUF668 and DUF3475 domain-containing protein (domain architecture ID 10571731)
DUF668 and DUF3475 domain-containing protein

Graphical summary Zoom to residue level show extra options »

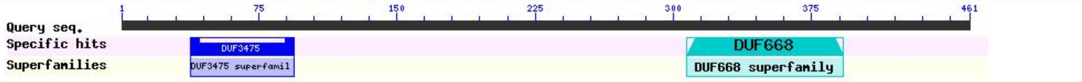

Search for similar domain architectures | Refine search

List of domain hits

Name	Accession	Description	Interval	E-value
[+] DUF668	pfam05003	Protein of unknown function (DUF668); Uncharacterized plant protein.	307-392	4.23e-43
[+] DUF3475	pfam11961	Domain of unknown function (DUF3475); This presumed domain is functionally uncharacterized. ...	38-94	1.92e-26

Ga_DUF668-16

Protein Classification

DUF668 and DUF3475 domain-containing protein (domain architecture ID 10571731)
DUF668 and DUF3475 domain-containing protein

Graphical summary Zoom to residue level show extra options »

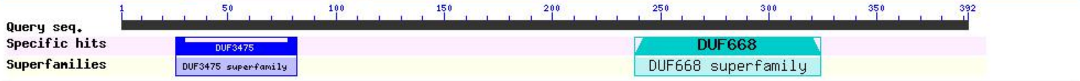

Search for similar domain architectures | Refine search

List of domain hits

Name	Accession	Description	Interval	E-value
[+] DUF668	pfam05003	Protein of unknown function (DUF668); Uncharacterized plant protein.	238-324	2.06e-42
[+] DUF3475	pfam11961	Domain of unknown function (DUF3475); This presumed domain is functionally uncharacterized. ...	26-82	9.13e-21

Ga_DUF668-17

Protein Classification

DUF668 and DUF3475 domain-containing protein (domain architecture ID 10571731)
DUF668 and DUF3475 domain-containing protein

Graphical summary Zoom to residue level show extra options »

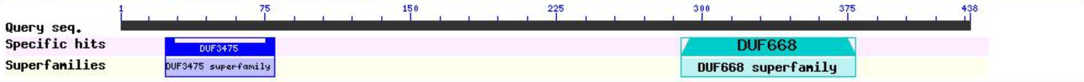

Search for similar domain architectures | Refine search

List of domain hits

Name	Accession	Description	Interval	E-value
[+] DUF668	pfam05003	Protein of unknown function (DUF668); Uncharacterized plant protein.	289-379	6.95e-30
[+] DUF3475	pfam11961	Domain of unknown function (DUF3475); This presumed domain is functionally uncharacterized. ...	24-80	2.18e-19

G. raimondii

Gr_DUF668-01

Graphical summary Zoom to residue level show extra options »

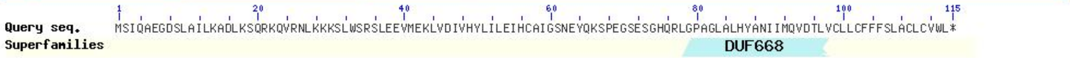

Search for similar domain architectures | Refine search

List of domain hits

Name	Accession	Description	Interval	E-value
[+] DUF668 super family	cl04883	Protein of unknown function (DUF668); Uncharacterized plant protein.	78-98	2.12e-09

Gr_DUF668-02

Protein Classification

DUF668 and DUF3475 domain-containing protein (domain architecture ID 10571731)
DUF668 and DUF3475 domain-containing protein

Graphical summary Zoom to residue level show extra options »

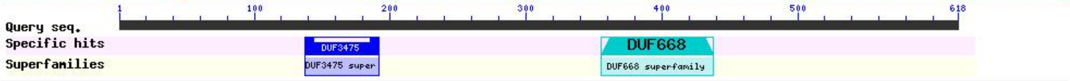

Search for similar domain architectures | Refine search

List of domain hits

Name	Accession	Description	Interval	E-value
[+] DUF668	pfam05003	Protein of unknown function (DUF668); Uncharacterized plant protein.	355-438	3.97e-41
[+] DUF3475	pfam11961	Domain of unknown function (DUF3475); This presumed domain is functionally uncharacterized. ...	137-192	3.76e-22

Gr_DUF668-03

Protein Classification

DUF668 and DUF3475 domain-containing protein (domain architecture ID 10571731)
DUF668 and DUF3475 domain-containing protein

Graphical summary Zoom to residue level show extra options »

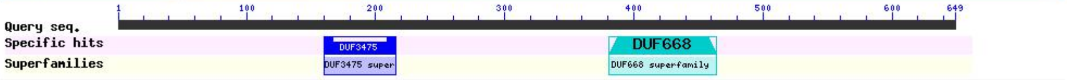

Search for similar domain architectures | Refine search

List of domain hits

Name	Accession	Description	Interval	E-value
[+] DUF668	pfam05003	Protein of unknown function (DUF668); Uncharacterized plant protein.	381-464	3.82e-47
[+] DUF3475	pfam11961	Domain of unknown function (DUF3475); This presumed domain is functionally uncharacterized. ...	160-216	5.37e-26

Gr_DUF668-04

Protein Classification

DUF668 and DUF3475 domain-containing protein (domain architecture ID 10571731)
DUF668 and DUF3475 domain-containing protein

Graphical summary

Zoom to residue level   show extra options »

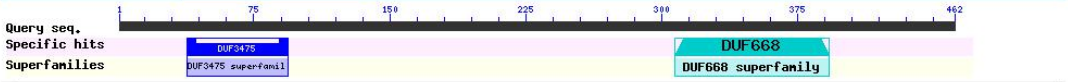

Search for similar domain architectures   Refine search

List of domain hits

Name	Accession	Description	Interval	E-value
[+] DUF668	pfam05003	Protein of unknown function (DUF668); Uncharacterized plant protein.	307-392	1.67e-44
[+] DUF3475	pfam11961	Domain of unknown function (DUF3475); This presumed domain is functionally uncharacterized. ...	38-94	2.93e-26

Gr_DUF668-05

Protein Classification

DUF668 and DUF3475 domain-containing protein (domain architecture ID 10571731)
DUF668 and DUF3475 domain-containing protein

Graphical summary

Zoom to residue level   show extra options »

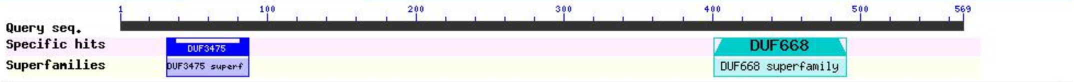

Search for similar domain architectures   Refine search

List of domain hits

Name	Accession	Description	Interval	E-value
[+] DUF668	pfam05003	Protein of unknown function (DUF668); Uncharacterized plant protein.	401-490	1.35e-44
[+] DUF3475	pfam11961	Domain of unknown function (DUF3475); This presumed domain is functionally uncharacterized. ...	32-87	2.66e-24

Gr_DUF668-06

Protein Classification

DUF668 and DUF3475 domain-containing protein (domain architecture ID 10571731)
DUF668 and DUF3475 domain-containing protein

Graphical summary

Zoom to residue level   show extra options »

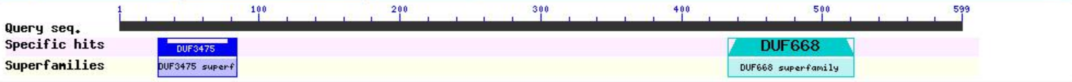

Search for similar domain architectures   Refine search

List of domain hits

Name	Accession	Description	Interval	E-value
[+] DUF668	pfam05003	Protein of unknown function (DUF668); Uncharacterized plant protein.	433-522	1.20e-48
[+] DUF3475	pfam11961	Domain of unknown function (DUF3475); This presumed domain is functionally uncharacterized. ...	28-84	1.28e-25

Gr_DUF668-07

Protein Classification

DUF668 and DUF3475 domain-containing protein (domain architecture ID 10571731)
DUF668 and DUF3475 domain-containing protein

Graphical summary

Zoom to residue level   show extra options »

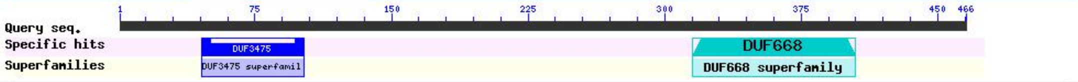

Search for similar domain architectures   Refine search

List of domain hits

Name	Accession	Description	Interval	E-value
[+] DUF668	pfam05003	Protein of unknown function (DUF668); Uncharacterized plant protein.	315-405	3.44e-30
[+] DUF3475	pfam11961	Domain of unknown function (DUF3475); This presumed domain is functionally uncharacterized. ...	46-102	1.12e-20

Gr_DUF668-08

Protein Classification

DUF668 and DUF3475 domain-containing protein (domain architecture ID 10571731)
DUF668 and DUF3475 domain-containing protein

Graphical summary

Zoom to residue level show extra options »

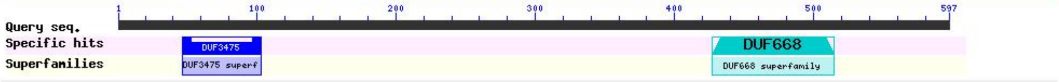

Search for similar domain architectures Refine search

List of domain hits

Name	Accession	Description	Interval	E-value
[+] DUF668	pfam05003	Protein of unknown function (DUF668); Uncharacterized plant protein.	427-514	3.79e-49
[+] DUF3475	pfam11961	Domain of unknown function (DUF3475); This presumed domain is functionally uncharacterized. ...	47-103	1.08e-23

Gr_DUF668-09

Protein Classification

DUF668 and DUF3475 domain-containing protein (domain architecture ID 10571731)
DUF668 and DUF3475 domain-containing protein

Graphical summary

Zoom to residue level show extra options »

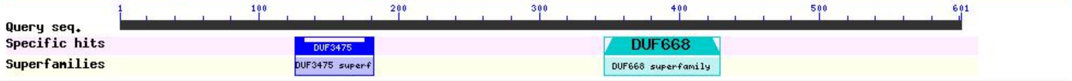

Search for similar domain architectures Refine search

List of domain hits

Name	Accession	Description	Interval	E-value
[+] DUF668	pfam05003	Protein of unknown function (DUF668); Uncharacterized plant protein.	346-429	2.34e-39
[+] DUF3475	pfam11961	Domain of unknown function (DUF3475); This presumed domain is functionally uncharacterized. ...	126-182	2.33e-22

Gr_DUF668-10

Protein Classification

DUF668 and DUF3475 domain-containing protein (domain architecture ID 10571731)
DUF668 and DUF3475 domain-containing protein

Graphical summary

Zoom to residue level show extra options »

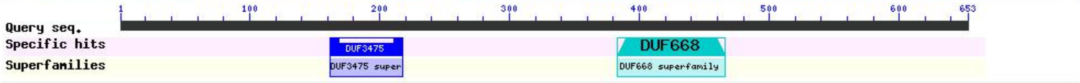

Search for similar domain architectures Refine search

List of domain hits

Name	Accession	Description	Interval	E-value
[+] DUF668	pfam05003	Protein of unknown function (DUF668); Uncharacterized plant protein.	383-466	3.76e-46
[+] DUF3475	pfam11961	Domain of unknown function (DUF3475); This presumed domain is functionally uncharacterized. ...	162-218	2.96e-23

Gr_DUF668-11

Protein Classification

DUF668 and DUF3475 domain-containing protein (domain architecture ID 10571731)
DUF668 and DUF3475 domain-containing protein

Graphical summary

Zoom to residue level show extra options »

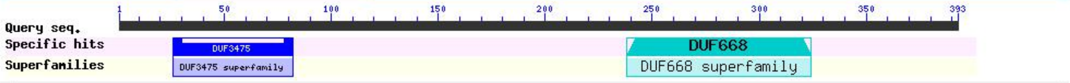

Search for similar domain architectures Refine search

List of domain hits

Name	Accession	Description	Interval	E-value
[+] DUF668	pfam05003	Protein of unknown function (DUF668); Uncharacterized plant protein.	238-324	5.30e-42
[+] DUF3475	pfam11961	Domain of unknown function (DUF3475); This presumed domain is functionally uncharacterized. ...	26-82	9.88e-21

Gr_DUF668-12

Protein Classification

DUF668 and DUF3475 domain-containing protein (domain architecture ID 10571731)
DUF668 and DUF3475 domain-containing protein

Graphical summary Zoom to residue level show extra options »

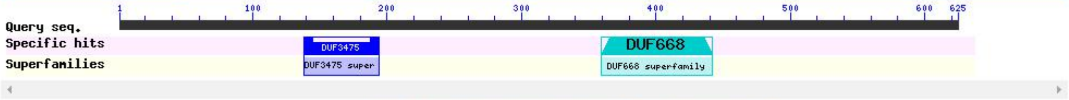

Search for similar domain architectures Refine search

List of domain hits

Name	Accession	Description	Interval	E-value
[+] DUF668	pfam05003	Protein of unknown function (DUF668); Uncharacterized plant protein.	359-442	7.74e-45
[+] DUF3475	pfam11961	Domain of unknown function (DUF3475); This presumed domain is functionally uncharacterized. ...	138-194	1.66e-22

Gr_DUF668-13

Protein Classification

DUF668 and DUF3475 domain-containing protein (domain architecture ID 10571731)
DUF668 and DUF3475 domain-containing protein

Graphical summary Zoom to residue level show extra options »

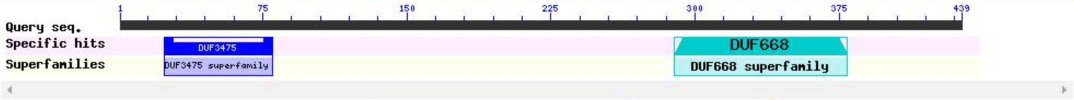

Search for similar domain architectures Refine search

List of domain hits

Name	Accession	Description	Interval	E-value
[+] DUF668	pfam05003	Protein of unknown function (DUF668); Uncharacterized plant protein.	289-379	6.16e-32
[+] DUF3475	pfam11961	Domain of unknown function (DUF3475); This presumed domain is functionally uncharacterized. ...	24-80	4.61e-20

Gr_DUF668-14

Protein Classification

DUF668 and DUF3475 domain-containing protein (domain architecture ID 10571731)
DUF668 and DUF3475 domain-containing protein

Graphical summary Zoom to residue level show extra options »

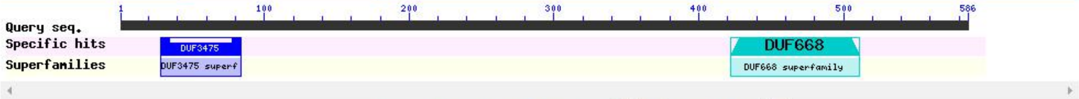

Search for similar domain architectures Refine search

List of domain hits

Name	Accession	Description	Interval	E-value
[+] DUF668	pfam05003	Protein of unknown function (DUF668); Uncharacterized plant protein.	422-511	7.04e-48
[+] DUF3475	pfam11961	Domain of unknown function (DUF3475); This presumed domain is functionally uncharacterized. ...	28-84	1.90e-24

Gr_DUF668-15

Protein Classification

DUF668 and DUF3475 domain-containing protein (domain architecture ID 10571731)
DUF668 and DUF3475 domain-containing protein

Graphical summary Zoom to residue level show extra options »

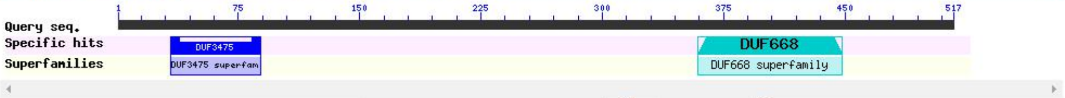

Search for similar domain architectures Refine search

List of domain hits

Name	Accession	Description	Interval	E-value
[+] DUF668	pfam05003	Protein of unknown function (DUF668); Uncharacterized plant protein.	359-448	8.57e-30
[+] DUF3475	pfam11961	Domain of unknown function (DUF3475); This presumed domain is functionally uncharacterized. ...	33-89	7.23e-19

Gr_DUF668-16

Protein Classification

DUF668 and DUF3475 domain-containing protein (domain architecture ID 10571731)
DUF668 and DUF3475 domain-containing protein

Graphical summary Zoom to residue level show extra options »

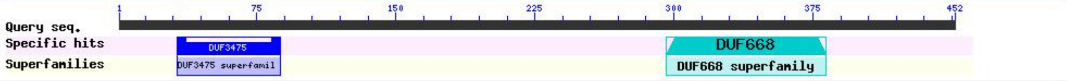

Search for similar domain architectures Refine search

List of domain hits

Name	Accession	Description	Interval	E-value
[+] DUF668	pfam05003	Protein of unknown function (DUF668); Uncharacterized plant protein.	296-382	7.22e-44
[+] DUF3475	pfam11961	Domain of unknown function (DUF3475); This presumed domain is functionally uncharacterized. ...	32-88	2.41e-23

Gr_DUF668-17

Protein Classification

DUF668 and DUF3475 domain-containing protein (domain architecture ID 10571731)
DUF668 and DUF3475 domain-containing protein

Graphical summary Zoom to residue level show extra options »

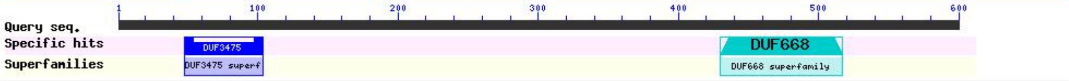

Search for similar domain architectures Refine search

List of domain hits

Name	Accession	Description	Interval	E-value
[+] DUF668	pfam05003	Protein of unknown function (DUF668); Uncharacterized plant protein.	430-517	3.54e-48
[+] DUF3475	pfam11961	Domain of unknown function (DUF3475); This presumed domain is functionally uncharacterized. ...	48-104	1.62e-23
