# Supplementary figures and images for: Genome-wide identification of the DUF668 gene family in cotton and expression profiling analysis of GhDUF668 in Gossypium hirsutum under adverse stress
Source: BMC Genomics. 2021 May 27;22:395. doi: 10.1186/s12864-021-07716-w (PMC8162019; doi:10.1186/s12864-021-07716-w)

Fig.S2 Conservative motifs of DUF668 gene family in *G. hirsutum*

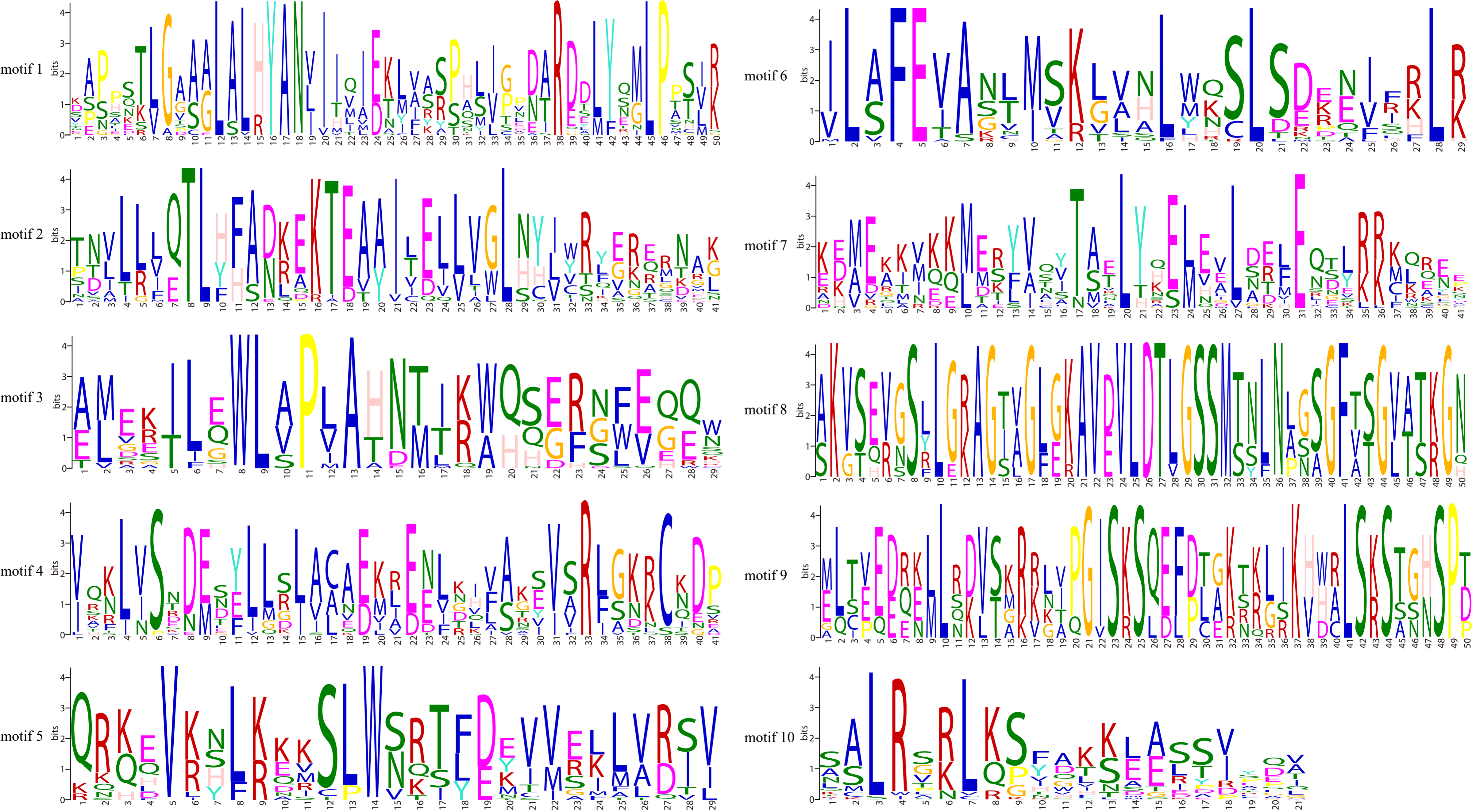

Supplement: Supplementary file 4 — Additional file 4: Figure S2. Conservative motifs of DUF668 gene family in Arabidopsis thaliana, rice, G. hirsutum, G. barbadense, G. arboreum and G. raimondii. [file 12864_2021_7716_MOESM4_ESM.pdf]

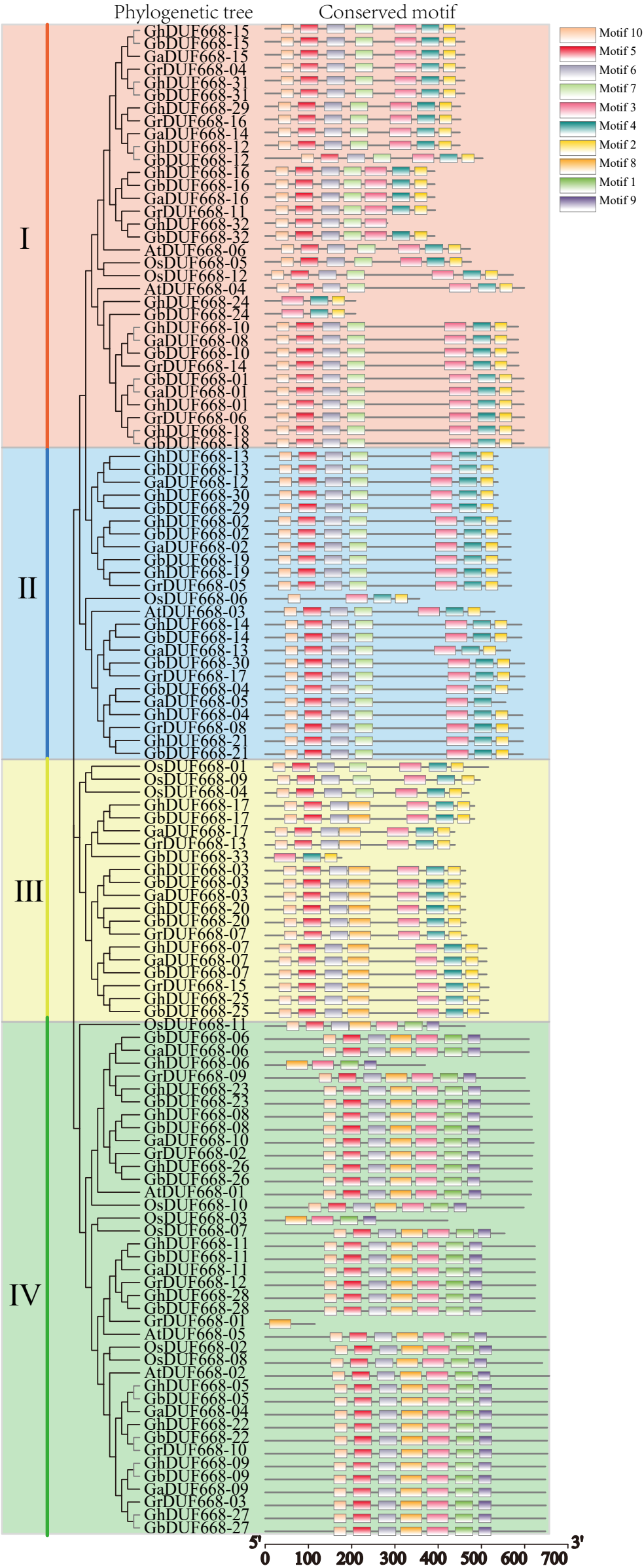

Supplement: Supplementary file 5 — Additional file 5: Figure S3. Conservative motifs of DUF668 gene family in G. hirsutum. [file 12864_2021_7716_MOESM5_ESM.pdf]

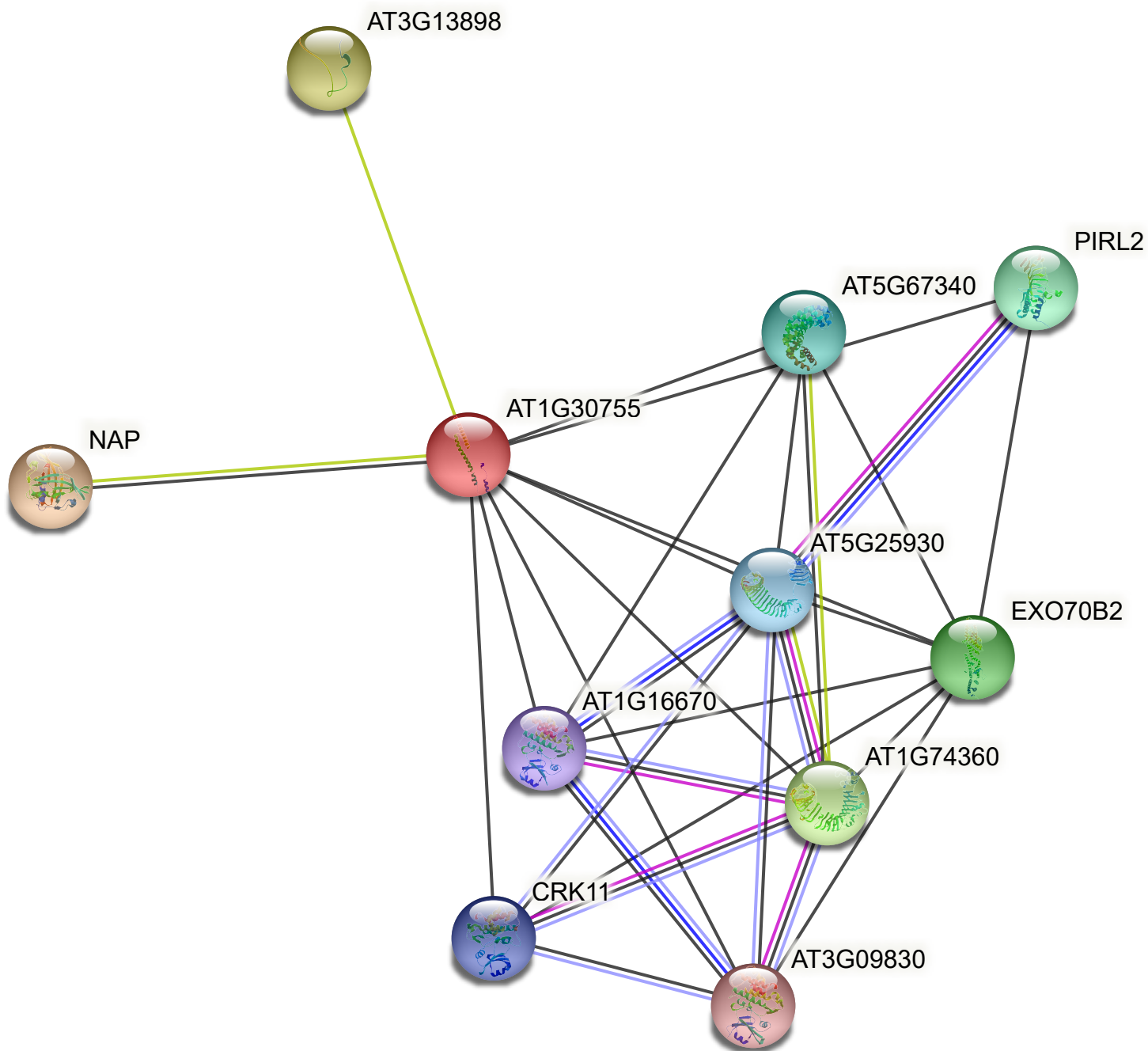

Supplement: Supplementary file 6 — Additional file 6: Figure S4. Interaction network of AT1G30755 proteins in Arabidopsis. [file 12864_2021_7716_MOESM6_ESM.pdf]
